# Reproducibility enhancement and differential expression of non predefined functional gene sets in human genome

**DOI:** 10.1186/1471-2164-15-1181

**Published:** 2014-12-24

**Authors:** Samoel RM da Silva, Gabriel C Perrone, João M Dinis, Rita MC de Almeida

**Affiliations:** Instituto de Física, Universidade Federal do Rio Grande do Sul, Av. Bento Gonçalves, 9500, 91501-970 Porto Alegre, RS Brazil; Instituto Nacional de Ciência e Tecnologia: Sistemas Complexos, Universidade Federal do Rio Grande do Sul, Av. Bento Gonçalves, 9500, 91501-970, Porto Alegre, RS Brazil

**Keywords:** Transcriptogram, Gene expression analysis, Transcriptome, Microarray

## Abstract

**Background:**

Transcriptogram profiling is a method to present and analyze transcription data in a genome-wide scale that reduces noise and facilitates biological interpretation. An ordered gene list is produced, such that the probability that the genes are functionally associated exponentially decays with their distance on the list. This list presents a biological logic, evinced by the selective enrichment of successive intervals with Gene Ontology terms or KEGG pathways. Transcriptograms are expression profiles obtained by taking the average of gene expression over neighboring genes on this list. Transcriptograms enhance reproducibility and precision for expression measurements of functionally correlated gene sets.

**Results:**

Here we present an ordering list for *Homo sapiens* and apply the transcriptogram profiling method to different datasets. We show that this method enhances experiment reproducibility and enhances signal. We applied the method to a diabetes study by Hwang and collaborators, which focused on expression differences between cybrids produced by the hybridization of mitochondria of diabetes mellitus donors with osteosarcoma cell lines, depleted of mitochondria. We found that the transcriptogram method revealed significant differential expression in gene sets linked to blood coagulation and wound healing pathways, and also to gene sets that do not represent any metabolic pathway or Gene Ontology term. These gene sets are connected to ECM-receptor interaction and secreted proteins.

**Conclusion:**

The transcriptogram profiling method provided an automatic way to define sets of genes with correlated expression, reduce noise in genome-wide transcription profiles, and enhance measure reproducibility and sensitivity. These advantages enabled biologic interpretation and pointed to differentially expressed gene sets in diabetes mellitus which were not previously defined.

**Electronic supplementary material:**

The online version of this article (doi:10.1186/1471-2164-15-1181) contains supplementary material, which is available to authorized users.

## Background

Genome-wide gene expression data are commonly obtained using microarrays, a current tool to assess cellular metabolism. There is already a wealth of gene expression data, related to an impressive number of experiments, that can be freely downloaded from public databases as, for example, the Gene Expression Omnibus - GEO [1] or ArrayExpress [2]. However, microarray data is considered to be very noisy and difficult to biologically analyze. There is plenty of literature [3–6] and initiatives [7–9] aiming at investigating data reliability and/or reproducibility. In this case, a method in genome scale that dampens the noise, preserves the signal, and provides a tool for biological interpretation is most welcome.

Gene Set Enrichment Analysis (GSEA) [10] is a powerful tool aiming at this purpose, and is designed primarily to compare gene expression of samples representing two different conditions. The first step of the method produces a list L by ranking the genes from the most to the least differentially expressed when comparing the gene expression for the different conditions. Then, considering a second gene list S, representing the genes of a particular pathway or functional assemblage of genes, the GSEA software measures the localization distribution of the S-genes in the complete list L. When this distribution is primarily found at the top or bottom of the list L, it is an indication that S as a whole is differentially expressed in the two conditions. An important GSEA feature is the possibility of assessing the significance that gene set S is differentially expressed, as indicated by this metric, as well as false discovery rates.

The GSEA analytical power resides on the fact that microarray data may be noisy. Hence, the average over a functional group of genes may enhance signal to noise ratio, provided the noise is random, and help biological interpretation [10]. In fact, GSEA has proven to discover new differentially expressed pathways in various diseases assays, where the individual genes are not extremely over or under expressed, but the expression of the whole set of genes represents a significant difference between conditions [10].

The transcriptogram, on the other hand, has been proposed as a method to analyze genome-wide gene expression data [11] and, similar to GSEA, considers the average of expression taken over a set of functionally related genes. One of the main differences, however, lays on the ways the gene sets are chosen. In the transcriptogram method, these sets are defined for each organism by running a window of a given size over an ordered gene list, whose ordering criterion is that the gene products are associated. The gene list covers all genes whose products have at least one protein-protein association in STRING database [12, 13] with a chosen confidence score. The result is a global view of the gene expression profile for each condition, which indicates the gene sets that are differentially expressed. There is not a previous selection for candidate pathways and it allows a significance assessment for these measures. As GSEA, the main goal of the transcriptogram is to provide hypotheses to biologically possible interpretation of the expression data, which renders particularly interesting the transcriptogram capability of offering an overall view, opening possibilities that have not been previously cogitated.

To build the ordered gene list necessary for transcriptograms, protein-protein association information is retrieved from STRING database to order the genes in such a way that the probability that any two gene products are associated exponentially decays with the distance between their positions on the list [11]. Protein-protein association in STRING database is strongly related to the probability that the associated proteins are listed in the same KEGG pathway [14], meaning that they collaborate in the same biological process. Consequently, nearby genes on the ordered list are expected to present correlated expressions. This fact is used to enhance both signal-to-noise and contrast-to-noise ratios and smoothen expression profiles by running box car averages over the ordered list, assuming that errors introduced by the assessment technique (microarrays, for example) are not correlated.

In this paper we start by discussing a generalized ordering method and its parameters and presenting the ordering for the human genome with a biological interpretation of its inherent logic. Then, by using publicly available data that considers technical replicates we show how contrast- to-noise ratio enhancement is attained. We then demonstrate that the transcriptogram enhances reproducibility of the measurements. We proceed by applying the method for a publicly available dataset focused on diabetes mellitus [15], and show how the transcriptogram method is able to point to differential expression of functionally related gene sets that may or not represent pathways or GO terms. Finally we discuss the advantages and limitations of the method and conclude.

## Methods

### The generalized ordering process

Transcriptograms are strongly focused on contrast-to-noise ratio enhancement in genome-wide expression measurements. This is achieved by first considering a genome-wide gene list and then, for each gene on the list, defining an interval made of 2*r* + 1 genes: the gene itself, its *r* neighbors to the left, and *r* neighbors to the right. Transcriptograms are then produced by assigning to each central gene the average expression level of its neighborhood on the list. These averages may dampen noise while preserving both signal and signal difference from the global average (contrast) when these neighboring genes have correlated expression: the gene ordering in the list is then very important.

The idea is to produce an ordered gene list such that the probability that any two genes are functionally associated exponentially decays with their distance on the list. Here gene association information, retrieved from STRING database [12, 13] considering all sources but text mining, comes in the form of a list of pairs of associated gene products. This information may be arranged as an adjacency matrix, *A*_*i*,*j*_, such that *A*_*i*,*j*_ = 1 whenever the products of the genes at positions *i* and *j* of the list are associated, and *A*_*i*,*j*_ = 0 otherwise. Observe that *A*_*i*,*j*_ depends on which proteins are occupying positions *i* and *j*, that is, depends on the way the list is ordered.

It is true that high gene expression levels do not necessarily imply high activity of the associated proteins: transcription data deals with gene transcripts, which may or may not be translated into proteins which, in their turn, may or may not be in an active state, depending on their phosphorylation or methylation configurations, for example. However, whole genome transcription measurements do provide valuable information about cellular metabolic state, and a high transcript level is a necessary condition for the production of the corresponding protein. Hence, it is reasonable to identify a protein list with the corresponding gene list or, for what regards this work, protein-protein association as information about gene-gene association.

To each gene/protein ordering, and its association matrix configuration, a cost function *F* may be defined as
1

This cost function has two terms:

-  |*i* - *j*|^*α*^, that depends on the distance (on the list) between the genes at positions *i* and *j*. For *α* > 0 and *A*_*i*,*j*_ = 1 (genes at positions *i* and *j* are associated), *F* decreases if *i* and *j* represent nearby positions;
-  The term in the brackets that increases when neighboring matrix elements are different. This term decreases when two genes that are associated to a third one are neighbors on the list.

Parameter *α* controls the strength of the first term. Results for *α* = 1 have been previously published, in an application to the cell cycle of *Saccharomyces cerevisae*[11]. Observe that only gene/proteins that present at least one protein-protein association will be considered in the ordering. Hence, for adjacency matrices retrieved from a protein-protein association database using different confidence scores, different orderings are obtained. In the examples we use in this paper we consider STRING confidence score 0.800, using version 9.05.

The ordering that minimizes *F* is performed by a Monte Carlo simulation [16]: at each computational step a pair of nodes is chosen and have their position swapped. The difference *ΔF* in the cost function is calculated and the change is accepted whenever *ΔF* ≤ 0 . In the case that the cost function is increased by *ΔF* > 0, the change is accepted with a probability proportional to , where *T* is a temperature-like parameter. Initially, *T* is taken as 0.01% of the initial value of the cost function, *F*, and at every 100 Monte Carlo Steps, the temperature is halved in a process known as simulated annealing, aiming at avoiding metastable states. The simulation ends when the number of changes has stabilized.

### Transcriptogram production

Transcriptomes for the osteosarcoma cell line 143B TK^-^ ρ_0_ and for the three cybrids were obtained from Gene Expression Omnibus database ( http://www.ncbi.nlm.nih.gov/geo/), under the accession GSE26244 [15].

Transcriptomes for five colorectal adenocarcinoma and matched normal tissues were obtained from Gene Expression Omnibus database, under the accession GSE41328 [17].

The samples were pre-processed using RMA [18], and the transcriptograms were produced using The Transcriptogramer, a software available at http://lief.if.ufrgs.br/pub/biosoftwares/transcriptogramer, using *α* = 1 and *r* = 80 (see Additional file 1: Figures S1-S3 and Additional file 1: Tables S1 and S2 on supplementary information online for *r* = 0 and *r* = 40).

### Statistical analysis for the transcription data

Considering transcriptogram levels, *P* -value comparisons between conditions were performed using a Welch’s two tailed *t*-test and, for a Bonferroni correction [19], we considered the number of gene positions in the ordering (9684) as the number of simultaneously tested hypotheses.

False discovery rates for each *P* -value were estimated as described in Refs. [20, 21].

## Results

In this paper we show that the transcriptogram method is able to point to functionally related gene sets that are differentially expressed between conditions. This is possible because the transcriptogram method enhances signal to noise ratio and gives a whole genome overview, that may be especially useful for a biological interpretation of gene expression data. In what follows we first produce an ordered list for human genes, as explained in materials and methods. Then, to illustrate the statistical fundaments for the transcriptogram method, we consider gene expression from different microarray experiments and calculate the variance stemming from the variability in the technique and then that originating in the biological variance. We proceed by obtaining the effect produced by box car averages in these variances, inherent to the transcriptogram method. We verify the calculated quantities by applying to microarray data retrieved from public databases. To demonstrate the advantages of the method, we apply to Hwang *et al.* data on diabetes mellitus [15] and identify new gene sets that are differentially expressed between conditions, providing significance assessments.

### Biological logic of the ordering

**Figure 1:**
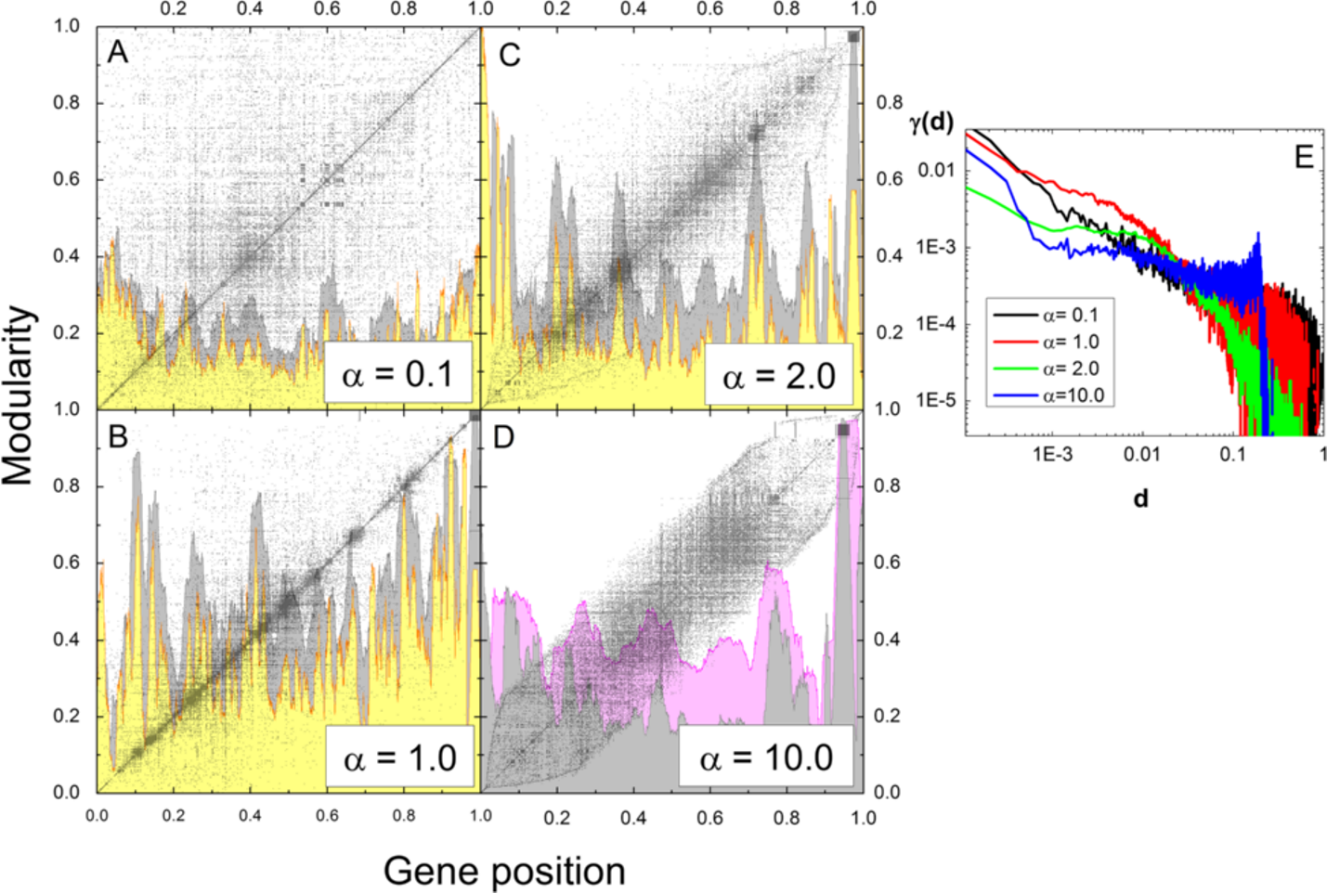
**Homo sapiens orderings for different values of** ***α*** **.** **A-D)** Adjacency matrix (black dots) for values of ***α*** as indicated in the legends. The axes have been normalized; the number of gene positions in each axis is 9684. The landscapes indicate the window modularity calculated with ***r*** = 80 (yellow), ***r*** = 200 (gray), and ***r*** = 1000 (magenta). Window modularity for ***r*** = 80 is not shown for ***α*** = 10.0 because it blurs the image. **E)** Probability of association between two genes separated by ***d*** positions on the ordered list, for different values of ***α***.

Figures 1 A-D present the adjacency matrix, *A*_*i*,*j*_, for *Homo sapiens* for different values of ordering parameter *α* (see Methods), where the dots represent protein-protein associations. Observe that while low values of *α* yield matrices with high concentration of dots near the diagonal, representing clusters of mutually interacting nodes, high values of *α* completely clean regions of the adjacency matrices representing interaction between genes that are distant on the ordered list. This observation is quantified by the occupation fraction *γ*(*d*), defined as
2

where *k*_*i*_ is the degree of the gene at position *i*, that is, the number of other genes that the *i*^th^ - gene is associated with. *γ*(*d*) gives the fraction of the *k*_*i*_ associated genes that are at a distance *d* (on the ordered gene list) from the *i*^th^ - gene, averaged over all genes. The plot of *γ*(*d*) versus *d* is presented in Figure 1E, for different values of *α*. Observe that, although for *α* ~ 1, *γ*(*d*) is higher for smaller *d*, for *α* ~ 10 the occupation fraction *γ*(*d*) suffers a sudden decrease at a distance of the order of 20% of the genome size. This fact will show to be especially important when choosing optimal transcriptograms, as explained further on in this paper.

Figures 1A-D present the window modularity *M*_*i*_(*r*) as colored background profiles, defined as
3

that gives the ratio between the number of associations between any two nodes inside the window of radius *r* around gene *i* to the number of associations that involve at least one gene in the window [11]. When *M*_*i*_(*r*) = 1 the genes inside do not interact with any genes outside the window, and when *M*_*i*_(*r*) = 0, the genes inside that window interact only with genes outside the window. In Figure 1A-D we present *M*_*i*_(*r*) for different values of *r* . Observe the peaks that correspond to clusters identified as dark blocks in the adjacency matrix plot. The high peak of the gray profile of Figure 1D located at the right end, for example, corresponds to genes belonging to the Gene Ontology (GO) term known as ‘Olfactory transduction’ that is annotated as being highly clustered [22].

**Figure 2:**
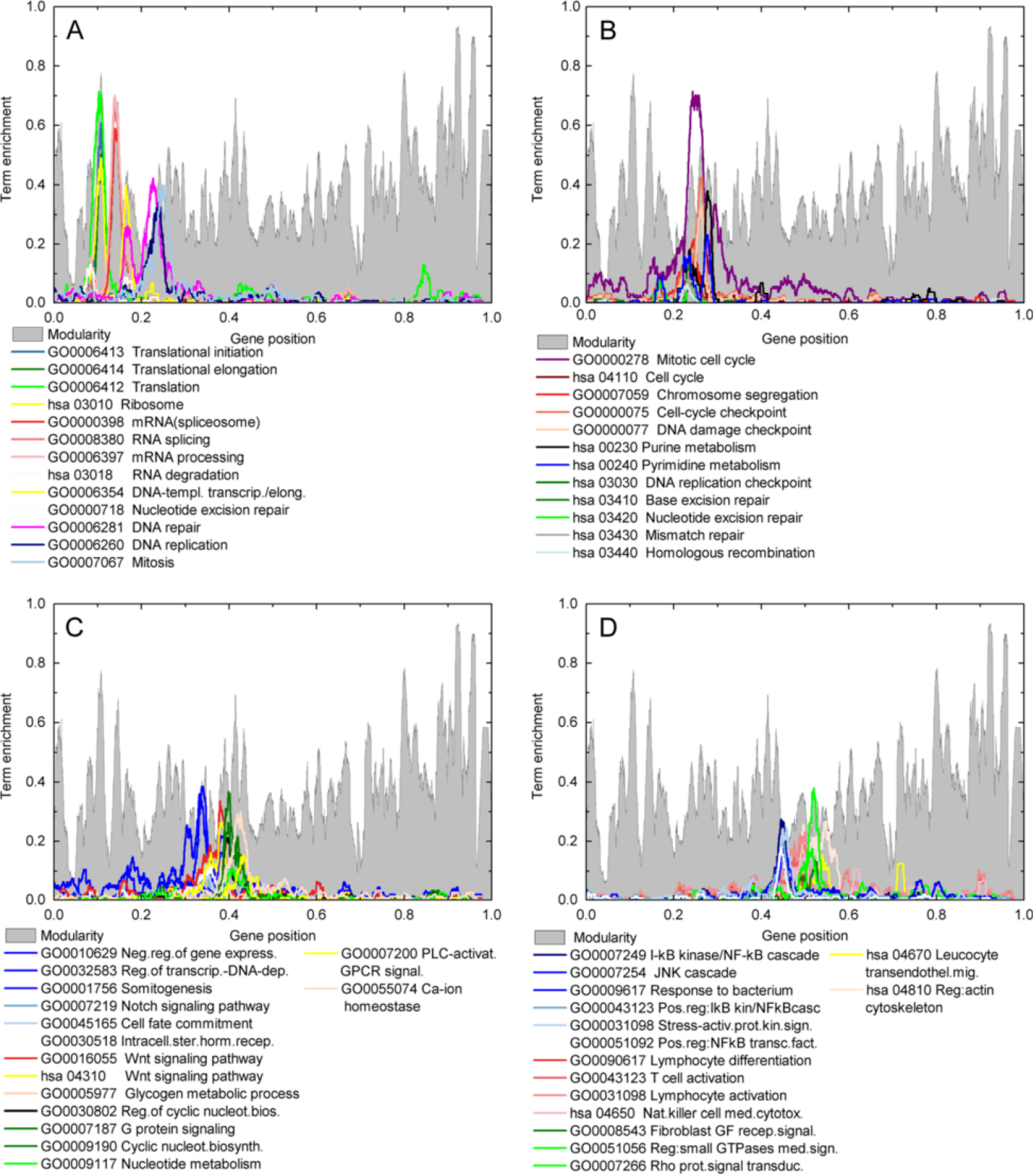
**Projection of Gene Ontology terms and KEGG pathways on the** ***α*** **= 1** **ordering and** ***r*** **= 80.** For **A)** genes associated with translation, RNA splicing, mitosis and DNA replication; **B)** genes associated with mitotic cell cycle, purine and pirimidine metabolism, and DNA repair pathways; **C)** genes associated with negative regulation of gene expression, to Notch and Wnt signaling pathways and to Calcium ion homeostasis; **D)** genes associated with the immune system, small GTP-ases mediated signaling and regulation of actin cytoskeleton.

**Figure 3:**
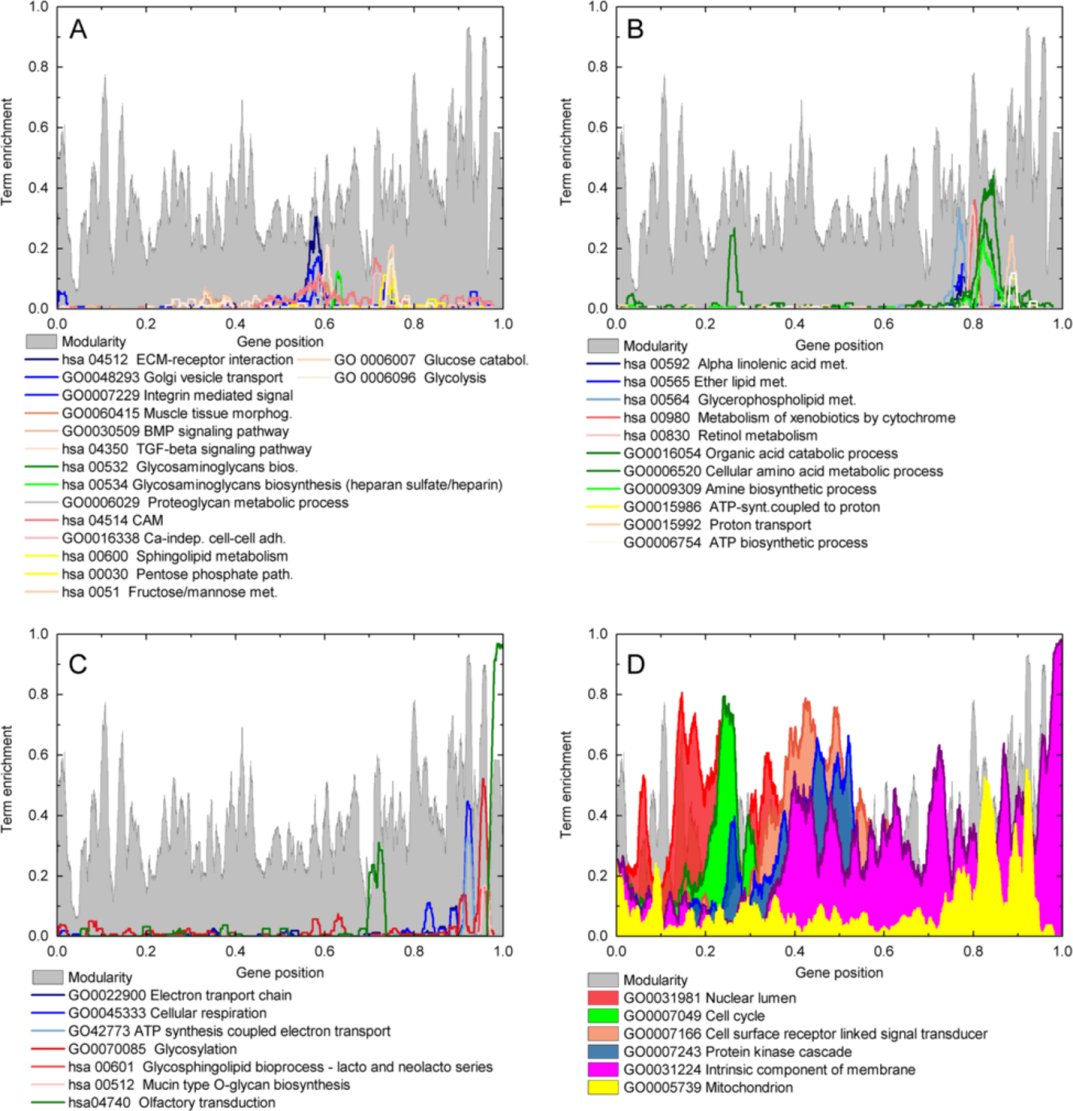
**Projection of Gene Ontology terms and KEGG pathways on the** ***α*** **= 1** **ordering and** ***r*** **= 80.** For **A)** genes associated with interaction with ECM, BMP and TGF-beta signaling pathways, cell adhesion, and glycolysis; **B)** genes associated with alpha-linolenic acid and retinol metabolism, cellular amino-acid metabolic process, proton transport and ATP biosynthetic process; **C)** genes associated to electron transport chain and respiration, glycosylation and olfactory transduction. **D)** Wider gene ontology terms profiles, summarizing the biological logic of the ordering.

Observe that protein-protein association STRING database integrates information from different databases, as KEGG, specific organism dedicated projects, results from high throughput experiments, etc. STRING ‘golden rule’ to define a confidence score for any association is related to the probability that the gene products co-participate in a KEGG pathway. As the information in the adjacency matrix has been retrieved from STRING, clusters reflect that neighboring genes on the list cooperate in biological functions. To evidence that, panels in Figures 2 and 3 present the density distribution of selected terms from the Gene Ontology (GO) database [23] and from KEGG pathways [14]. Each term profile is obtained by first retrieving from GO or KEGG database the list of genes of a given term, then assigning to each gene on the ordered list the value 1 or 0 depending on whether or not the gene is listed as a component of the term or pathway [11]. Then, for each central gene of windows of size 2*r* + 1, the term/pathway profile intensity is defined as the average over the window. The result is a smooth profile for each term whose peaks indicate the regions of the ordered list enriched with genes of that term. Observe that various peaks in modularity may be identified to some class of pathway or ontology terms.

The ordering in Figures 2 and 3 has been obtained for *α* = 1.0 while the profiles were built considering *r* = 80. The reasons for choosing each value are interconnected and depend on the association matrix properties presented in Figure 1, and on the purposes of the data analysis. In one hand, transcriptograms may be used for discriminate two or more conditions in a large set of samples, aiming at a diagnostic classification. In this case, the criterion for choosing *α* and *r* is based on the classification power of the method. Alternatively, data analysis may focus on finding both differentially expressed gene sets as well as a biological explanation for the differences in the samples phenotypes. The first case we address elsewhere; here we chose to approach the second possibility.

KEGG pathways, which are built based on biochemical reactions and may provide biological explanations for phenotype variation, present the order of 100 genes, which corresponds to roughly *d* = 0.01 in Figure 1E (9684 genes times 0.01). In that region, *γ* is maximum for *α* = 1.0, that is, this value of *α* optimizes the clustering of groups of the order of 100 genes. We calculated the modularity profile for *α* = 1.0 and different window sizes. Figure 1B shows two examples, *r* = 80 and *r* = 200. Decreasing windows radius may reduce peaks height, since it can increase the number of connections with gene/proteins outside the windows. For transcriptogram production, it would imply not considering the expression of correlated genes/proteins in the same window average. On the other hand, increasing windows size may merge separated pathways in the same window, which may also decrease the signal. By comparing the yellow and gray profiles, it is possible to verify that the yellow peaks height is smaller for gene position around 0.5, while it is larger around gene position 0.9, indicating in this last case that the window size could be further reduced. Hence, the optimal window size for *α* = 1.0 depend on the position on the ordering. This is so probably for the different evolutionary origins of the term/pathways enriching in each region, as presented in Figures 2 and 3, where we chose *r* = 80.

The profiles shown in Figures 2 and 3 indicate the localization on the ordered list of ontology terms and metabolic pathways, which follows a biological logic. These figures change depending on the organism and on the ordered list. They do not depend, however, on a specific gene expression experiment. Figure 2A shows ontology terms and pathways profiles that are concentrated on the beginning of the list and are related to translation of mRNA into proteins, represented by the peaks in different shades of green (individual labels are presented in the figure legend). Moving on the ordering list to the right there appear peaks in shades of red, related to RNA processing, and then by blue profiles associated to DNA processing. Not surprisingly, the last profile in this figure, represented by a light blue, corresponds to the gene ontology term associated to Mitosis. Figure 2B continues the saga presented in 2 A. A broader profile is shown in purple, corresponding to the ontology term Mitotic cell cycle, which is a larger Gene Ontology term. In the same interval, smaller profiles are shown, all corresponding to different biological processes related to cell cycle, including DNA repair and checking mechanisms. Figure 2C presents the profiles associated with cell fate commitment and other related terms or pathways, as Notch, Wnt, or Somitogenesis terms. Figure 2D marks the interval of genes involved in immune response and the associated reorganization of actin cytoskeleton.

Figures 3A, 3B and 3C proceed in recognizing the biological functions of each interval of the ordering list. In particular, Figure 3A presents two different intervals: the first one describes terms related to the extra cellular matrix (ECM) and integrin mediated signaling. The second interval, to the right, represented by shades of salmon and yellow, are related to cellular adhesion molecules and pathways. The interval corresponding to positions between 0.65 and 0.7 is enriched with molecules associated to the membrane, secretion or interaction with the exterior of the cell. However, in spite of the high window modularity peak (followed by a valley), we could not find a GO term or KEGG pathway that would also have its most representative peak in this region, as indicated by a functional annotation analysis using David Functional tools. Figure 3B focuses on terms and pathways linked to signal transduction and gene expression regulation as ether lipid metabolism and retinol metabolism pathways, respectively. Then, continuing to the right on the ordered list, there appears peaks related to glycolysis, that is, energy metabolism. Figure 3C presents peaks in blue and red which are related to ATP proton transport and to glycosylation. The highest green peak, located at the extreme right interval of the ordered list in this figure, is related to olfactory transduction pathway and presents a smaller portion, located in the same region as the cellular adhesion and membrane associated molecules, indicating the membrane associated molecules of the olfactory transduction pathway.

Figure 3D presents a summary of the biological organization of the ordered gene list, by showing broader gene ontology profiles: the list begins with genes related to the nuclear lumen, followed by genes involved in cell cycle, then cell surface and signal transduction and finally intrinsic components of membrane overlapped by mitochondrion associated genes.

### Transcriptograms for technical and biological replicates

Transcriptograms are produced for each sample by first assigning to each gene its transcription level as measured from a microarray experiment, after a data pre-processing as, for example, Robust Microarray Average (RMA) method [18]. Next, considering a window of size *w* = 2*r* + 1 centered at each gene on the ordered list and assigning to this central gene the window average over the transcription levels. The transcriptogram is the resulting profile, which may be plotted in a graph that gives the window average transcription level as a function of the position of the window central gene on the list.

Suppose that the pre-processed (normalized) transcriptome data in an experiment with *n* replicates is given by , with *i* = 1, ⋯, *N*, *b* = 1, ⋯, *n*_*b*_ and *a* = 1, ⋯, *n*_*a*_ where *b* and *a* label, respectively, biological and technical replicates, *n*_*b*_ and *n*_*a*_ are the number of biological and technical replicates, summing up to *n*_*b*_ × *n*_*a*_ transcriptomes for each experimental condition labeled by subscript *k*. Finally, *i* is the gene position on the oredered list, and *N* is the number of genes/proteins present in the ordering (that is, that appear in the protein-protein association data used to build up the adjacency matrix).  is assumed to have three additive components:
4

where (*s*_*i*_)_*k*_ is the expected signal for the gene located at position *i* of the ordering under the experimental condition *k*, and hence we dropped the indices *b*, *a*.  responds for the biological variation and should depend on the experimental conditions and on the biological replicate, but not on the technical replicate. Finally,  is a stochastic noise, which varies from a measurement to another. Considering a pre-process protocol using RMA method, the values are given as logarithm base 2 of the measured intensity. The addition in Eq.(4) represents hence multiplicative effects. A transcriptogram  for these data is produced by taking the average of the expression levels over a window of radius *r* on the ordered list, that is,
5

where *θ*_*j*_ = 1 if the gene at position *j* is a target for some probe set of the microarray platform used to generate the transcriptome, and *θ*_*j*_ = 0 otherwise. Observe that  when all genes inside the window are represented in the microarray platform.

As an application of the method, we consider the interesting experiment presented by Hwang *et al.*[15] focused on finding the effect of different haplogroups on cybrids with the same nuclear DNA. For this, they hybridized osteosarcoma cells, depleted from mitochondria and without thymidine kinase activity (143 TK^-^ ρ_0_ cell line) with mitochondria from different donors presenting either N9a (3 donors), F (5 donors), and D5 (4 donors) haplogroups. For each different donor haplogroup, 4 technical replicate transcriptomes were produced, summing up, respectively, 12, 20 and 16 transcriptomes. For the 143 TK^-^ ρ_0_ cell line 6 technical replicated transcriptomes were produced.

**Figure 4:**
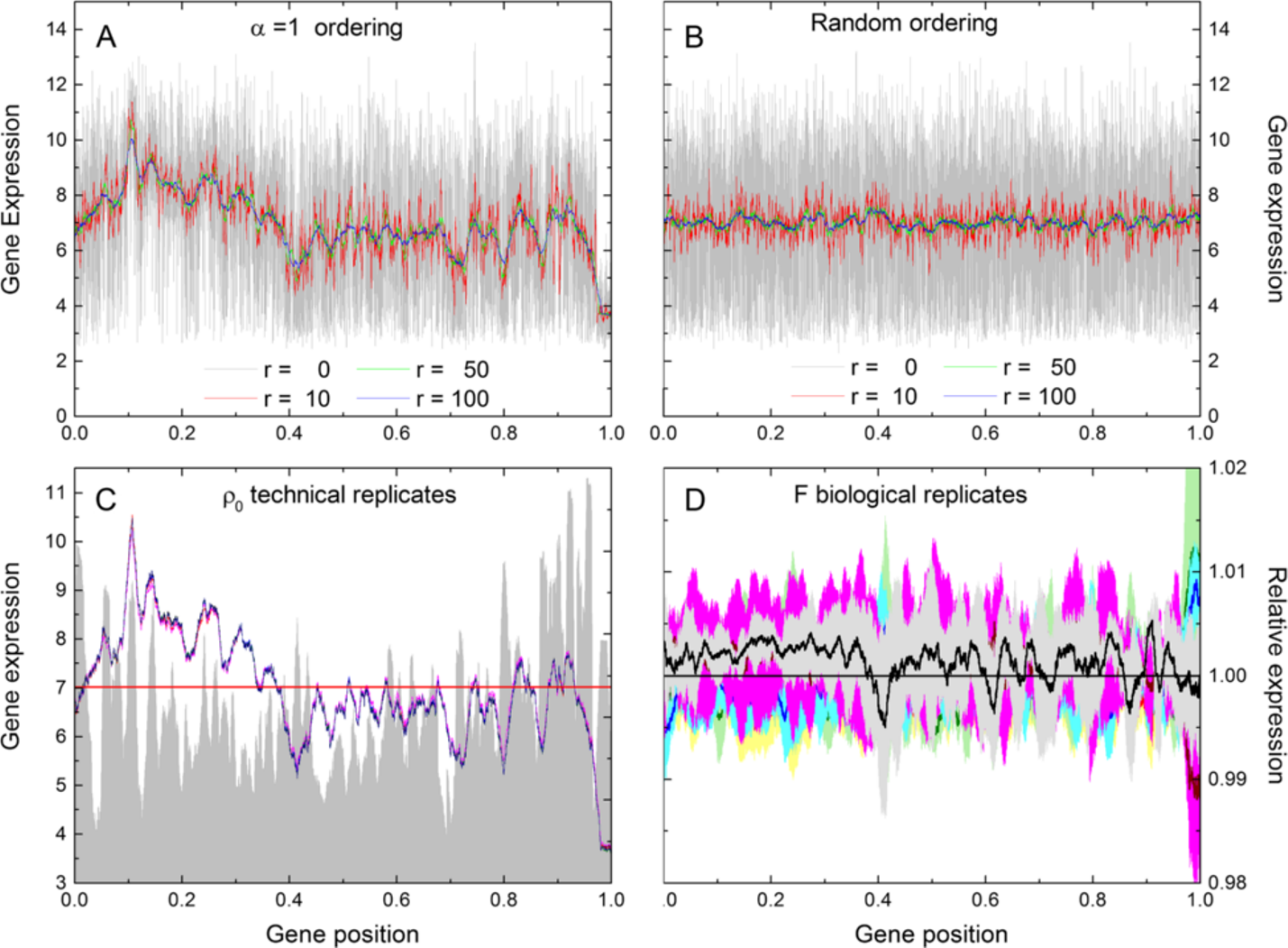
**Transcriptograms for technical and biological replicates with different windows sizes.** Transcriptograms of technical replicates using a **A)** *α* = 1 ordered gene list and **B)** a randomly ordered gene list. Observe that both profiles are smoothened by window averages, indicating noise reduction. The transcriptogram obtained for the ordered list, however, conserves signal as compared to that a random ordered list is used. **C)** Transcriptograms for *α* = 1 and *r* = 80 for the six technical replicates of ρ_0_ cell line with modularity (*r* = 80) as the gray profile in the background. The horizontal red line is the expression global average. Observe that the trasncriptograms overlap almost perfectly. **D)** Average transcriptograms for *α* = 1 and *r* = 80 over sets of four technical replicates for each one of the five F cybrid biological replicates, relative to the average transcriptogram of all F cybrids (represented by the horizontal line at relative expression equal to 1). The colored shadows around each profile correspond to standard deviations. Observe the change in scale: the standard deviations are of the order of 1% of the average expression level. Since all colored regions overlap, the set of all 20 transcriptograms (biological and technical replicates) are not significantly different.

Figures 4A and 4B show examples of transcriptograms with different window sizes for one sample of ρ_0_ cell line. Figure 4A shows the transcriptogram of one replicate with different window sizes for genes ordered as explained above with *α* = 1.0, while Figure 4B shows the same data as a transcriptogram calculated using a randomly ordered gene list. The gray profiles, for *r* = 0, show the transcription data, pre processed with RMA, before transcriptogram window averages. Observe that the transcriptograms are smoother as the window size increases in both figures, but in Figure 4A the transcriptograms deviate more from their global average, presenting larger fluctuations, while in Figure 4B the profiles fluctuate with a smaller amplitude: the contrast between the global average and local deviations are preserved in Figure 4A. It implies that transcriptograms calculated over ordered lists are more capable of discerning differences in expression in different intervals of the list, as compared to those calculated over randomly ordered lists. Furthermore, Figures 4C and D show that these fluctuations are a characteristic of each sample condition. Figure 4C presents the transcriptograms for *r* = 80 for the 6 ρ_0_ technical replicates, with the gray background representing the window modularity, also for *r* = 80. The horizontal, red line represents the global average of the transcriptograms. The profiles coincide almost perfectly, meaning that the expression levels they express are common to all technical replicates. Figure 4D compares biological and technical replicates belonging to the same class. For each biological replicate of F cybrids, we define a relative average transcriptogram, , as the mean over the technical replicates divided by the average over all F cybrid transcriptograms, that is
6

The standard deviations considering a set of technical replicates for each biological replicate is represented in Figure 4D as the colored region following each line. Observe that the standard deviations are of the order of 1% of the average level , represented by the black horizontal line. All colored regions overlap, indicating that, as far as these transcriptograms are concerned, there are not significant differences in expression among the biological replicates sets. This trend is also present for the other two cybrids N9a and D5 that were analyzed by Hwang and collaborators. Additional file 1: Figure S4 in supplementary information online presents the expression data after RMA pre-processing and before the window averages for a small interval of the ordering list (this is observed for all intervals): the variation in expression between the technical replicates is small as compared to the variation between expression of the different genes, indicating that microarray data measurement errors are not the main variation source for transcriptograms. Together, Figure 4 show that i) window averages reduces noise of one sample by smoothening the profiles (4 A and B), ii) the contrast to global average is more reduced for the random list, iii) transcriptograms for technical replicates overlap almost perfectly while still showing marked differences from the global average (4 C), and iv) biological replicates are not significantly different (4 D).

To compare conditions, a pertinent quantity to estimate the significance of a difference between means is the ratio (*ϵ*_*i*_)_1,2_ given as
7

where (.) is the average over the replicates, and  is the standard deviation for gene expression in the window around the *i* -th gene on the ordered list. The corresponding expression for the transcriptogram values, (Z_*i*_)_1,2_, is
8

where
9

with  and  being the variances due to, respectively, the measurement stochastic noise and the biological difference between samples under the same experimental conditions.  is the average of the covariance for the biological variation over all pairs of genes inside the window. The biological meaning of this last term may be clarified by the consideration of two limit possibilities:  when biological samples have the expression of the genes inside the window varying independently of one another, and  when the biological variations are the same for all genes inside the window, that is,  for all pairs (*j*, *j*^'^). Details of this calculation are found in Supplementary Information online.

In Eqs. (7) and (8), the difference between means is given by the numerator and the variances sum, by the denominator. Consequently transcriptograms may enhance the sensitivity of microarray measures provided that  decreases less than the denominator in Eq.(8). This may happen when the biological variance is not correlated over the window, that is, when
10

for both conditions *k*, which yields
11

For the transcriptogram procedure to enhance sensitivity for difference between means,  should decrease slower than , that is, slower than  for windows where all genes are represented in the microarray. This behavior may not happen for different reasons: i) the conditions do not present differences in expression for the genes in that window, this is the trivial case when the means are indeed the same; ii) the difference in the samples causes some genes to increase and others to decrease their expression, what should be expected in case the window randomly mixes genes. In fact this is what happens when a random ordering is considered: the transcriptogram difference between means is strongly reduced. Even in case the window comprises genes belonging to the same pathway or biological function, it still can happen that the increase in expression of some genes compensates the decrease of others and the difference between means could be more strongly dampened than the random case. In this situation, the transcriptogram method causes a loss of information. To evaluate which is the general effect of the transcriptogram, we define a contrast-to-noise ratio as follows.

We start by analyzing the effects of technical noise in data sets produced by Hwang and collaborators [15]. We define the contrast  for class *k* as
12

that gives the absolute difference at each gene position from the condition average transcriptogram to the condition global average. A related quantity, defined as , is used in the literature as an estimative of the signal in large data sets. As we show in what follows,  yields a more stringent test for the transcriptogram performance. The noise is taken as the standard deviation of the transcriptograms at each gene position, such that the contrast-to-noise ratio, *ω*^*k*^(*r*), is defined as
13

while signal-to-noise ratio, , is defined as
14

Observe that signal-to-noise ratio gives the average for transcriptogram values in units of transcriptogram standard deviation, while contrast-to-noise ratio estimates the average of the difference of transcriptogram values to the global average in units of transcriptogram standard deviation. As window radius increases, there is a loss of contrast but not necessarily of signal. In fact,  is a monotonically increasing function of the window radius *r*, while *ω*^*k*^(*r*) must decrease as *r* → *N*, since all transcriptogram values go to the global average in this limit, as shown in Additional file 1: Figure S5 in supplementary materials online. In what follows we use contrast-to-noise ratio to assess the transcriptogram performance as an method to analyze microarray data.

**Figure 5:**
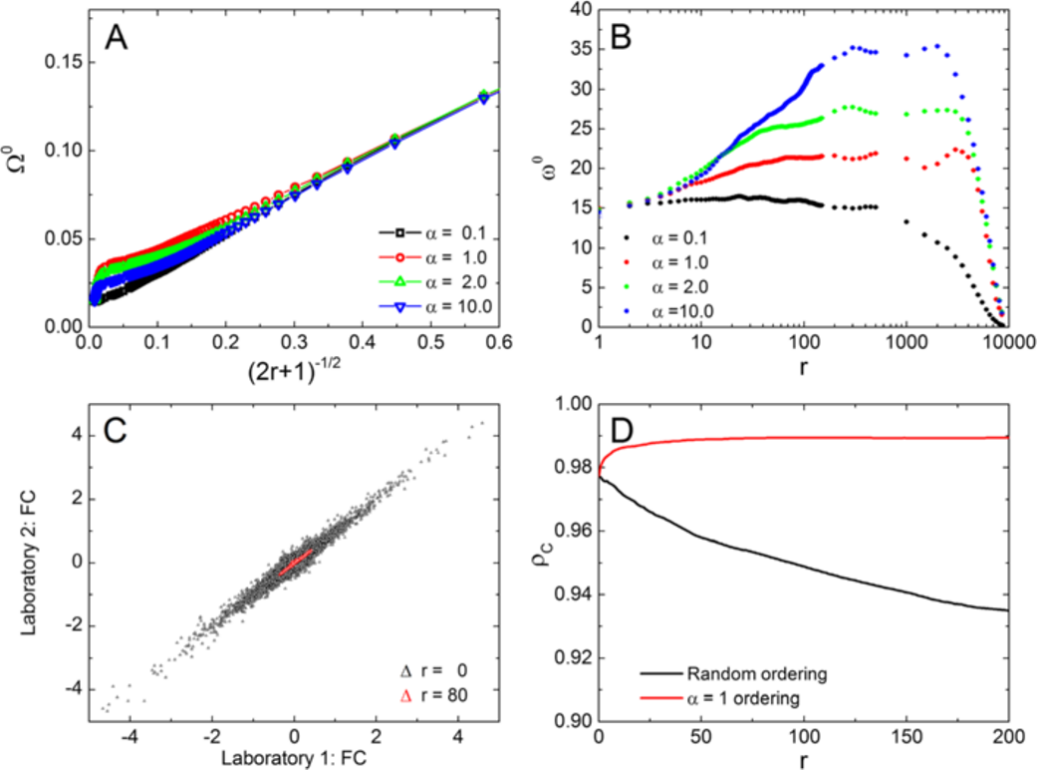
**Noise reduction and signal enhancement on transcriptograms as function of window sizes.** **A)** Average noise Ω^0^ as a function of the square root of window size,  for different values of *α*, calculated for the of ρ_0_ cell line. **B)** Signal to noise ratio *ω* ^0^ as a function of window radius *r*, for different values of *α*, in a linear-log plot, calculated for ρ_0_ cell line. **C)** Concordance between laboratories, with relative expression (Fold Change) as metric. Each axis corresponds to the fold change value for each gene obtained by one laboratory. **D)** Concordance correlation coefficient, *ρ*_*C*_, versus window radius, *r*, for the *α* = 1 - ordered (red symbols) and random ordered (black symbols) gene list. Observe that *r* = 0 corresponds to the analysis for transcriptomes.

In Figure 4C, for each gene position *i*,  is the mean distance from the transcriptogram profiles to the horizontal red line representing the global average. , on the other hand, may be estimated from the small width of the set of the 6 transcriptogram profiles. Figure 5A presents the plot of the average noise, Ω^0^(*r*), for ρ_0_ technical replicates, defined as

15

as a function of , the inverse of the square root of window size, for different values of *α* : for all values of *α*, Ω^0^(*r*) goes as , as it should be expected for a random noise. This behavior is also observed for the other sets of technical replicates (data not shown). In Figure 5B we present the signal to noise ratio *ω*^0^(*r*) as a function of the window radius *r*: *ω*^0^(*r*) initially increases with *r* and, for *α* ≥ 1, we may state that the data processing method implied by the transcriptogram significantly enhances contrast-to-noise ratio. *ω*^0^(*r*) decreases for window sizes comparable to the whole genome due to the lowering of the signal term, that is, when window size approaches genome size, transcriptogram values approaches the global average of transcription levels. Surprisingly, *α* = 10 presents the highest contrast-to-noise ratio enhancement. Nevertheless *α* = 1 was chosen in the example presented in section 2.2: biological interpretation is facilitated due to a more adequate clustering of GO terms and KEGG pathways. Additional file 1: Figure S6 in supplementary materials online shows the same GO terms profiles as in Figure 2A, but for an ordered list obtained with *α* = 10 : the peaks in both window modularity and GO term profiles are greatly reduced, meaning that for each GO term the genes are spread over the ordered list and a difference in expression in a given interval of the list comprises genes participating in different biological functions.

A serious consequence of noisy measurements is the deleterious effect on the reproducibility of measurements by different scientific teams. As microarray measurements are noisy, reproducibility of such measurements deserves a careful attention. In particular, a MAQC Project challenge to the community [8] has focused on the determination of differentially expressed genes (DEGs) when measuring samples in different conditions. A gene is indicated as a DEG when its relative average expression exceeds a pre-determined statistics metric such as a *P* -value or fold-change (FC).

Assume, for example, that two conditions (A and B) have been compared in an experiment, with a number *n* of biological replicates. To find the differentially expressed genes, the relative average expression (over the replicates) for each gene, or fold change (FC) , is calculated for every gene *i*. When this relative expression exceeds a pre-determined fold change value, considered as a metric of the desired statistical significance, the gene is said to be differentially expressed under the different conditions. A second laboratory performs the same experiment and obtains another list of relative average expression values. The data from both laboratories may be displayed in a scatter plot in log-log scale, where each dot represents one gene, whose coordinates are the relative average expressions obtained by each laboratory. When the laboratories agree for a gene, the corresponding dot is located very near the diagonal, since both coordinates are similar. Points with coordinates near (0, 0) have their relative average expression near 1 for both laboratories, meaning that both laboratories agree that the expression is not different under conditions A and B. Points that deviate from the diagonal represent a significant disagreement.

Figure 5C shows the relative average expression for the experiment reported by Lin *et. al.*[17], where microarray data have been generated from five matched colorectal adenocarcinoma and normal tissues. The same material has been processed for hybridization in two different laboratories. The values in the abscissa and ordinate axes for each dot in Figure 5C correspond to the relative expression of a gene, , obtained by each laboratory. Black symbols correspond to transcriptome data (*r* = 0) and red ones, to the relative average transcriptogram values  for *r* = 80. As red dots are consistently located nearer the diagonal, Figure 5C shows that the use of the transcriptogram values enhances reproducibility as compared to using transcription values as they are produced after the pre-processing method (RMA, in this case). On the other hand, as the red dots are nearer the center point, (0, 0), also the contrast has been reduced. These figures show the results only for windows radius *r* = 80. To investigate how reproducibility behaves with the transcriptogram window radius, we calculated the concordance correlation coefficient, *ρ*_*c*_[16], defined as
16

where indices 1 and 2 discriminate between laboratories and, for *ℓ* = 1, 2,
17

is the average of the relative expression of the *ℓ* -th laboratory,
18

is the standard deviation of the relative average expression levels, and
19

is the covariance between laboratories. Observe that transcriptograms taken over windows of radius *r* = 0 correspond to transcriptomes. Figure 5D presents the dependence of the concordance correlation coefficient, *ρ*_*c*_, given in Eq.(16), as a function of the transcriptogram radius *r* for different ordered lists, for the same data as before. The same concordance coefficients for transcriptograms have also been calculated for random orderings: in this case the contrast-to-noise ratio is not enhanced and, consequently, does not improve reproducibility.

### Finding differentially expressed gene sets: Hwang et al data

**Figure 6:**
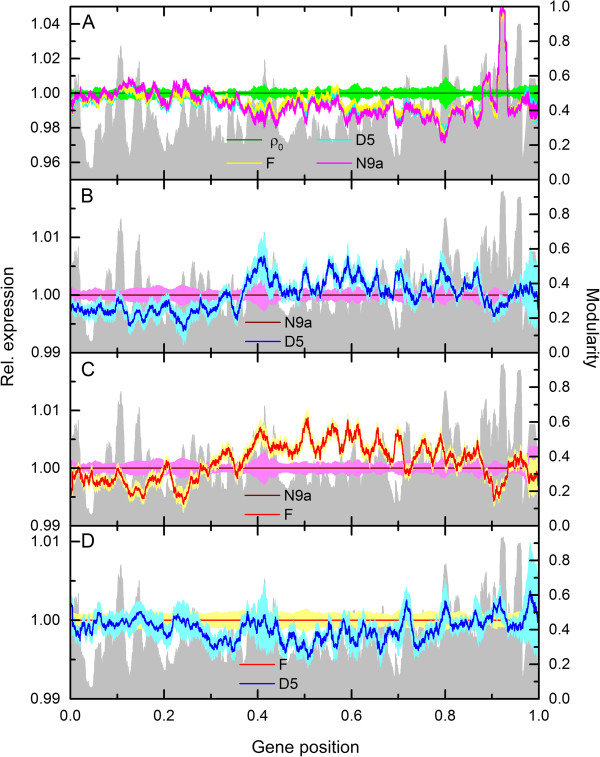
**Relative average transcriptograms (** ***α*** **= 1,** ***r*** **= 80) with colored regions standing for the respective standard errors.** For **A)** N9a, F and D5 cybrids relative to the ρ0 cell line; **B)** D5 cybrid relative to N9a cybrid transcriptograms; **C)** F cybrid relative to N9a cybrid; **D)** D5 cybrid relative to F cybrid.

Figure 6A presents the average transcriptogram of each cybrid cell line for the Hwang *et al.* data relative to the average transcriptogram of 143 TK^-^ ρ_0_ cell line, where we used *α* = 1 and *r* = 80. The olive green horizontal line stands for the 143 TK^-^ ρ_0_ cell line average transcriptogram and the green region comprehends the points where the deviation from this average is less than one standard error. The pink, yellow and cyan fat lines are the average transcriptograms for each cybrid class; lines width being the respective standard errors. The gray background is the window modularity, as presented in Figures 2 and 3.

The transcriptograms in Figure 6A indicates that N9a cybrids (magenta) are more different from the 143 TK^-^ ρ_0_ samples than F or D5 cybrids, as previously pointed by Hwang and collaborators [15]. Figures 6B and C present the average transcriptograms of, respectively, D5 and F relative to N9a cybrids (horizontal line). Again, the lines correspond to the average transcriptograms, while the colored regions following the lines stand for the standard error of each class. Although both D5 and F follow the same trend, there are some regions of the ordering where F average is farther from N9a average, also in agreement with Hwang *et al.*[15]. Finally, Figure 6D presents D5 cybrid average transcriptogram relative to F average, represented by the horizontal line, again with the colored regions around the lines being the respective standard errors. There are few intervals where the colored regions do not overlap, but for a small gap. Additional file 1: Figure S2, in supplementary materials online presents a version of Figure 6 for *r* = 40. The trend is the same.

**Figure 7:**
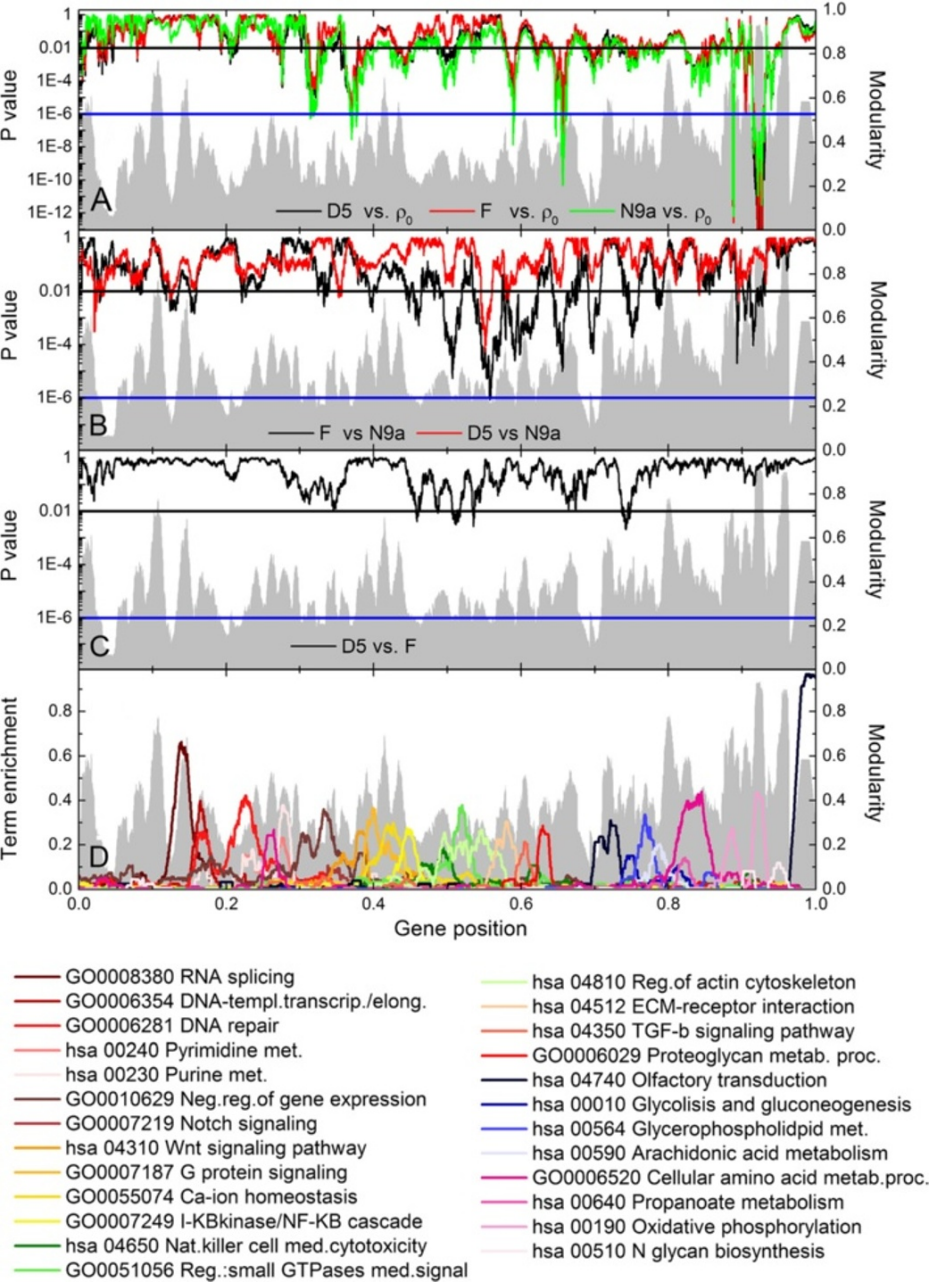
***P*** **-values of transcriptogram means for all gene positions in** ***α*** **= 1** **ordering and** ***r*** **= 80.** For **A)** F, D5 and N9a cybrids compared to ρ0 cell line; **B)** F and D5 cybrids compared to N9a cybrid; **C)** and D5 cybrid compared to F cybrid. **D)** Selected gene ontology terms and KEGG pathways profiles. Gray background is the window modularity profiles, to guide the eye. The horizontal, black line in panels **A-C** represents ***P*** = 0.01, while the horizontal blue line at P = 0.01/9684 corresponds to the Bonferroni correction for multiple hypotheses testing.

To estimate the significance of the differences between the means in Figure 6, we run a two tailed Welch’s *t* test and assigned a *P* -value for every point in the transcriptograms, as shown in Figure 7A-C. We also run a False Discovery Rate testing (FDR). The blue, horizontal lines in Figure 7A-C correspond to Bonferroni corrected *P*_*B*_ = 0.01 values (= 0.01/9684 ~ 10^- 6^), which is intended to correct for the simultaneous tests of 9684 hypotheses corresponding the number of gene positions on the ordered list. The black, horizontal lines stand for *P* = 0.01 values and the corresponding FDR values for each class comparison is given in Table 1: FDR < 0.06 (6%) at *P* = 0.01 for the comparison between any cybrid against ρ_0_ cell line (Figure 7A), FDR < 0.28 at *P* = 0.01 for the comparison of D5 against N9a cybrids (Figure 7B), FDR < 0.04 at *P* = 0.01 for the comparison of F against N9a cybrids (Figure 7B), and finally FDR < 0.33 at *P* = 0.01 for the comparison of D5 against F cybrids. The lower panel, in Figure 7D, gives the profiles of selected GO terms and KEGG pathways, overlaying the modularity gray background, to guide the eye. It can be observed in Figure 7A that, as it would be expected from previous results [15], the extremely low *P* -values for the three cybrid conditions appear at gene positions enriched with Oxidative Phosphorylation pathway (around gene position ~ 0.92), corresponding to the highest peaks in Figure 6A. Additional file 1: Figure S3 in supplementary materials online repeats Figure 7 for *r* = 40.

**Table 1:** False discovery rate (FDR) calculated at different *P* -values for *α* = 1 ordering and *r* = 80 transcriptograms for different pairs of conditions

	***FDR***					
P-value	N9a vs. ρ0	F vs.ρ0	D5 vs.ρ0	F vs.N9a	D5 vs.N9a	D5 vs. F
**0.1**	0.145389	0.171001	0.139366	0.157776	0.295737	0.548948
**0.05**	0.091211	0.110416	0.080086	0.098791	0.269759	0.464294
**0.01**	0.038148	0.056859	0.025014	0.038567	0.278242	0.326013
**0.005**	0.025381	0.045119	0.016431	0.025939	0.27055	0.617382
**0.001**	0.01015	0.024616	0.005609	0.009346	0.161983	---

## Discussion

Even under the stringent Bonferroni test, the transcriptogram analysis indicate various regions where cybrids differ from ρ_0_ cell line, besides the expected Oxidative Phosphorylation region around gene position 0.92, as shown in Figure 7A. These regions correspond to gene positions around 0.315, associated with regulation of gene expression; around 0.375, associated with Wnt signaling and Insulin signaling pathways; around 0.5905, associated with secreted proteins in the extracellular part or involved in ECM-receptor interaction; and around 0.65, an interval enriched with proteins associated with the immune system and Sema domain. Coherently all these pathways or GO terms have been linked to Type II diabetes. Mitosis and cell cycle play an important role in the development of the disease [25, 26], the relation between Wnt and Type II diabetes is discussed in references [27–29], the disruption of ECM in diabetic kidneys and vascular system is well known (see, for example, references [30–32]), Sema domains are present in some insulin receptors (HGF, MSP) [33], while the connection between the immune system and diabetes is reviewed in [34]. The differential expression unveiled by transcriptogram method makes biological sense and gives a global view of the main metabolic disruptions of the disease. Furthermore, the regions on the ordering associated to ECM-receptor represent functionally related gene sets that are not representing some specific GO term or metabolic pathway, as indicated by a functional annotation analysis using David Functional Annotation Tools [24, 35]. Lowering the scores, but still in a very conservative limit, some gene sets are indicated as differentially expressed with *P* < 0.01 and FDR < 0.06. We first point to the upside down peaks around gene positions 0.19 and 0.21, and 0.27, associated with, respectively, chromosome organization, DNA metabolic processes and mitotic cell cycle. As the cybrids have all the same nuclear DNA content as ρ_0_ cell line, this significant differential expression could be attributed to the different mitochondrial DNA. Other significant expression regions, around positions 0.31 and 0.37, are related to regulation of transcription and Wnt signaling, while around positions 0.5 and 0.58 are associated with transmembrane processes as transmembrane receptor protein tyrosine kinase signaling pathway and ECM-receptor interaction or cellular adhesion. Finally, at the right end of the ordering, Figure 7A shows the deep upside peaks related to mitochondria activity, around positions 0.83, 0.89, and 0.92. GSEA and Gene Trail analyses, as reported by Hwang *et al.*[15], have not pointed out these significant alterations.

Figure 7B focus on the comparison of D5 and F against N9a cybrids. As should be expected, the differences are smaller; they are however highly significant, as indicated by both *P* values and FDR tests. In agreement with GSEA analysis by Hwang and collaborators [15], the comparison against N9a cybrids points to stronger differences in F than in D5 expression profile. One gene set is indicated as significant after applying Bonferroni correction to *P* -values: around gene position 0.557 a set of 146 genes is found to be differentially expressed (the black F-cybrid profile touches the horizontal, blue line). It corresponds to one point in the transcriptogram, which represents an average over 2*r* + 1 = 161 positions. However, some of the genes are not targets for any probe in the microarray chip. This gene set is associated with wound healing and blood coagulation GO terms. Again, other gene sets may be spotted as differentially expressed, with lower scores. Around gene positions 0.12 and 0.15, upside down peaks crossing the black horizontal line at *P* = 0.01 indicate differentially expressed gene sets linked to RNA processing and Spliceosome while at positions 0.33 and 0.35 the upside down peaks are related to transcription regulation. Farther to the right, transcriptograms relative to N9a cybrids show stronger alterations for F cybrids than for D5. These alterations are in the ordering regions enriched with genes associated with either response to wounding and regulation of cytokine production or to signals linked to some receptor at cell membrane or secreted proteins acting in the extracellular space. Finally, to the right end, some upside down peaks indicate significant differences of expression for energy metabolism, as genes related to mitochondrion, respiratory chain and oxidative phosphorylation, what could be attributed to the different DNA content of the haplogroups.

Figure 7C presents the relative transcriptogram of D5 against F cybrids. As already pointed out, these cybrids are more similar. Nevertheless, some difference can be spotted, with *P* < 0.01. However, FDR for these findings are of the order of 0.33, so they should be considered only as possible hypotheses to be further investigated.

(Additional file 2) File Ordering.txt in supplementary material online provides the gene ordered list together with the respective *P* -values for all gene positions and each comparison between conditions, while (Additional file 3) DifferentiallyExpressedGeneSets.txt provides the gene set corresponding to each upside down, significant peak in Figure 7.

In what regards the other relative transcriptograms presented in supplementary materials online, namely, for *r* = 40 (Additional file 1: Figure S2 and Additional file 1: Figure S3) and *r* = 0 (Additional file 1: Figure S1) on *α* = 1 ordered list and for *r* = 80 on a randomly ordered list (Additional file 1: Figure S7), we found the following. *r* = 0 reflects an analysis without a box car average of transcriptome data and without a fold change or *P* -value cutoff; while there seems to be many significant points, it is hard to interpret what is going on without some kind of clustering procedure. *r* = 40 represents a viable alternative to *r* = 80; both analyses present the same trend and basically the same conclusions. However, the tuning of the best window size may depend on the interval of the ordering with the most interesting findings. The random ordering for *r* = 80 is also interesting: there are more peaks than in *α* = 1 ordering, but with less significant *P* -values. Again, it is more difficult to biologically interpret the results.

Finally, we remark that the transcriptogram analysis provides a hierarchical assessment of gene expression data, starting from a global view of the metabolic differences, then indicating the most altered pathways, and finally pointing to the genes participating in these alterations, as provided by the files in supplementary materials online. A more careful analysis of these findings, aiming at a deeper biological interpretation would be interesting, but is beyond the scope of this paper.

## Conclusion

In this paper we discussed the limits for which the transcriptogram method for analyzing genome-wide gene expression measurements is capable of enhancing the contrast-to-noise ratio and facilitate biological interpretation of the data. When this is the case, transcriptograms offer the possibility of a precise expression measurement for functionally correlated gene sets and, consequently, increase reproducibility between laboratories. Moreover, although these gene sets may be enriched with genes belonging to some Gene Ontology term, KEGG pathway or other previously annotated gene sets, the transcriptograms results concern to automatically defined gene sets, based on the probability that their products are associated as listed in STRING database. Consequently, gene sets different from those previously considered may be discovered as differentially expressed between conditions.

The contrast-to-noise ratio enhancement is obtained through a box car average over a gene list that is ordered such as to approximate biologically related genes. This is accomplished using STRING database for protein-protein association to build an adjacency matrix and a Monte Carlo simulation to order the gene list. This is not the only way to obtain a functionally ordered gene list, and it may not be the optimal. Certainly an investigation to determine the optimal ordering for finding differentially expressed gene sets would be interesting. Some of this work is now in progress [36] and will be published elsewhere.

Gene expression averages over functionally related genes as a way to improve the precision of expression measurements without losing a biological sensible interpretation have also been proposed by Gene Set Enrichment Analysis (GSEA) [10]. As pointed there, these methods increase reproducibility between laboratories and allow assessing moderate differences in sets of many genes that can be producing important effects in the cellular phenotype. Transcriptograms, however, provide further a global view of the differentially expressed sets of functionally related genes, independently of previous hypotheses, representing a possible source for new ideas on what is modified when comparing different conditions. An example for diabetes mellitus was discussed where the transcriptogram method clearly indicated pathways not linked to the energy metabolism to be differentially expressed in different cybrids, as for example, sets of genes acting in the extracellular space or linked ECM-receptor interaction, or in wound healing and blood coagulation.

We finally remark that transcriptogram method is applicable to any whole genome gene expression measurements, independently of platform or technology (it certainly applies to RNASeq data, for example). The transcriptogram may also be used for diagnostic purposes, where the transcriptogram data are fed to machine learning algorithms. In this case, where precision in sample classification is favored to biological interpretation, higher values of *α* are generally more efficient. These applications are being considered as now and the results will be published elsewhere. Also, a software devised to produce functionally ordered gene lists, together with transcriptogram production is freely available at http://lief.if.ufrgs.br/pub/biosoftwares/transcriptogramer.

### Availability of supporting data

The supporting data have been previously published and made available by other authors, as cited in the text.

## Electronic supplementary material

Additional file 1: **daSilvaSupplementary.docx: file containing details on calculations and two figures and two tables as described in the main text.** (DOCX 2 MB)

Additiojnal file 2: **Ordering.txt: a ten columns text file, where the columns give, respectively, the position in the ordering list, the Gene name at that position, the ENSEMBL ID for the protein associated to gene in second column, the relative gene position, the P-value for the comparisons between pairs of conditions, as the header indicate.** (TXT 792 KB)

Additional file 3: **DifferentiallyExpressedGeneSets.txt: a text file with the genes in each differentially expressed set for each comparison between pairs of conditions.** (TXT 593 KB)

## Abbreviations

GO: Gene ontology
ECM: Extra cellular matrix
GEO: Gene expression omnibus
GSEA: Gene set enrichment analysis
RMA: Robust microarray average
T2DM: Type 2 diabetes mellitus
mtDNA: Mitochondrion DNA
MAQC: Micro array quality control
DEG: Differentially expressed genes
FC: Fold change
FDR: False discovery rate.
