# Supplementary material for: Reproducibility enhancement and differential expression of non predefined functional gene sets in human genome
Source: BMC Genomics. 2014 Dec 24;15(1):1181. doi: 10.1186/1471-2164-15-1181 (PMC4326474; doi:10.1186/1471-2164-15-1181)
Supplement: Supplementary file 1 — Additional file 1: daSilvaSupplementary.docx: file containing details on calculations and two figures and two tables as described in the main text. (DOCX 2 MB) [file 12864_2014_6993_MOESM1_ESM.docx]

Supplementary Information for

Reproducibility enhancement and differential expression of non predefined functional gene sets in human genome

Samoel R. M. da Silva^1^, Gabriel C. Perrone^1^, João Dinis^1^, and Rita M.C. de Almeida^1,2^

^1^Instituto de Física and ^2^Instituto Nacional de Ciência e Tecnologia: Sistemas Complexos, Universidade Federal do Rio Grande do Sul

Av. Bento Gonçalves, 9500

91501-970 Porto Alegre, RS, Brazil

**I - Statistical Analysis in detail**

Suppose that the pre-processed transcriptome data in an experiment with $n$ replicates, is given by $\left( t_{i}^{b,a} \right)_{k}$, with $i=1,\cdots,N$ , $b=1,\cdots,n_{b}$ and $a=1,\cdots,n_{a}$ where $b$ and $a$ label, respectively, biological and technical the replicates,$n_{b}$ and $n_{a}$ are the number of biological and technical replicates, summing up $n_{b}\times n_{a}$ transcriptomes for each experimental condition, labeled by subscript $k$. Finally, $i$ is the gene position on the ordered list, and $N$ is the number of genes/proteins present in the ordering (that is, that appear in the protein-protein association data used to build up the adjacency matrix). $\left( t_{i}^{b,a} \right)_{k}$ is assumed to have three additive components:

$$\left( t_{i}^{b,a} \right)_{k}=\left( s_{i} \right)_{k}+\left( v_{i}^{b} \right)_{k}+\left( \rho_{i}^{b,a} \right)_{k}, (S1)$$

where $\left( s_{i} \right)_{k}$ is the expected signal for the gene located at position $i$ of the ordering under the experimental condition $k$, and hence we dropped the indices $b,a$. $\left( v_{i}^{b} \right)_{k}$ responds for the biological variation and should depend on the experimental condition and on the biological replicate, but not on the technical replicate. Finally, $\left( \rho_{i}^{b,a} \right)_{k}$ is a stochastic noise, which varies from a measurement to the other? Considering a pre-process protocol using RMA (Irizarry 2003), the values are given as logarithm base 2 of the measured intensity. The additions in Eq.(S1) represent hence multiplicative effects. A transcriptogram $\left( T_{i}^{b,a} \right)_{k}$ for these data is produced by taking the average of the expression levels over a window of radius $r$ on the ordered list, that is,

$$\left( T_{i}^{b,a} \right)_{k}=\frac{1}{\sum_{j=i-r}^{i+r} \theta_{j}}\sum_{j=i-r}^{i+r} \left[ \left( s_{j} \right)_{k}+\left( v_{j}^{b} \right)_{k}+\left( \rho_{j}^{b,a} \right)_{k} \right]\theta_{j}, (S2)$$

where $\theta_{j}=1$ if the gene at position $j$ is a target for some probe set of the microarray platform used to generate the transcriptome, and $\theta_{j}=0$ otherwise. Observe that $\sum_{j=i-r}^{i+r} \theta_{j}=2r+1$ when all genes inside the window are represented in the microarray platform. We define the transcriptogram values for the signal,$\left( S_{j} \right)_{k}$, biological variation, $\left( V_{j}^{b} \right)_{k}$ and stochastic noise, $\left( R_{j}^{b,a} \right)_{k}$, as

$$\begin{matrix} \left( S_{j} \right)_{k} & = & \frac{1}{\sum_{j=i-r}^{i+r} \theta_{j}}\sum_{j=i-r}^{i+r} \left( s_{j} \right)_{k}\theta_{j} \\ \left( V_{j}^{b} \right)_{k} & = & \frac{1}{\sum_{j=i-r}^{i+r} \theta_{j}}\sum_{j=i-r}^{i+r} \left( v_{j}^{b} \right)_{k}\theta_{j} \\ \left( R_{j}^{b,a} \right)_{k} & = & \frac{1}{\sum_{j=i-r}^{i+r} \theta_{j}}\sum_{j=i-r}^{i+r} \left( \rho_{j}^{b,a} \right)_{k}\theta_{j} \end{matrix} . (S3)$$

Microarray experiments are commonly intended to detect variations in gene expression levels due to some variation of the experimental conditions. These variations may be due to different sample preparation, cell cycle stage, cell lines, tissues, or disease states. In all cases the variation is assessed by comparing the transcriptome data, which may or not have been adequately averaged. The basic idea is to assess the differences and their statistical significance. To show the enhancement that transcriptograms may bring to this process, we first calculated the variance between replicates for $\left( t_{i}^{b,a} \right)_{k}$ and $\left( T_{i}^{b,a} \right)_{k}$, that is

$$\left\langle\left( t_{i}^{b,a} \right)_{k}^{2} \right\rangle-\left\langle\left( t_{i}^{b,a} \right)_{k} \right\rangle^{2}= \left[ \left\langle\left( v_{i}^{b} \right)_{k}^{2} \right\rangle-\left\langle\left( v_{i}^{b} \right)_{k} \right\rangle^{2} \right]+\left[ \left\langle\left( \rho_{i}^{b,a} \right)_{k}^{2} \right\rangle-\left\langle\left( \rho_{i}^{b,a} \right)_{k} \right\rangle^{2} \right] + 2 \left[ \left\langle\left( v_{i}^{b} \right)_{k} \left( \rho_{i}^{b,a} \right)_{k} \right\rangle-\left\langle\left( v_{i}^{b} \right)_{k} \right\rangle\left\langle\left( \rho_{i}^{b,a} \right)_{k} \right\rangle\right] (S4)$$

where $\left\langle\cdot\right\rangle=\frac{1}{n_{a}}\sum_{a=1}^{n_{a}} \frac{1}{n_{b}}\sum_{b=1}^{n_{b}} \cdot$ is the average over the replicates. The first term in the right-hand side of Eq.(7) is the variance due to the biological difference between samples under the same experimental condition or conditions, $\left( \sigma_{i}^{\text{bio}} \right)_{k}^{2}$ , the second term is the variance due to the measurement stochastic noise, $\left( \sigma_{i}^{\text{tech}} \right)_{k}^{2}$ , and the third term is the covariance of the two effects. Here we assume this covariance to be zero, that is

$$\left( \sigma_{i}^{\text{total}} \right)_{k}^{2}=\left\langle\left( t_{i}^{b,a} \right)_{k}^{2} \right\rangle-\left\langle\left( t_{i}^{b,a} \right)_{k} \right\rangle^{2}=\left( \sigma_{i}^{\text{bio}} \right)_{k}^{2}+ \left( \sigma_{i}^{\text{tech}} \right)_{k}^{2} . (S5)$$

The variance of the transcriptogram, on the other hand, is given by

$$\left\langle\left( T_{i}^{b,a} \right)_{k}^{2} \right\rangle-\left\langle\left( T_{i}^{b,a} \right)_{k} \right\rangle^{2}$$

$$= \left[ \left\langle\left( V_{i}^{b} \right)_{k}^{2} \right\rangle-\left\langle\left( V_{i}^{b} \right)_{k} \right\rangle^{2} \right]+\left[ \left\langle\left( R_{i}^{b,a} \right)_{k}^{2} \right\rangle-\left\langle\left( R_{i}^{b,a} \right)_{k} \right\rangle^{2} \right] , (S6)+ 2 \left[ \left\langle\left( V_{i}^{b} \right)_{k} \left( R_{i}^{b,a} \right)_{k} \right\rangle-\left\langle\left( V_{i}^{b} \right)_{k} \right\rangle\left\langle\left( R_{i}^{b,a} \right)_{k} \right\rangle\right]$$

that can be written in terms of $\left( \sigma_{i}^{\text{bio}} \right)_{k}^{2}$ and $\left( \sigma_{i}^{\text{tech}} \right)_{k}^{2}$ since

$$\left[ \left\langle\left( V_{i}^{b} \right)_{k}^{2} \right\rangle-\left\langle\left( V_{i}^{b} \right)_{k} \right\rangle^{2} \right]= \frac{\bar{\left( \sigma_{i}^{\text{bio}} \right)_{k}^{2}}}{\sum_{j=i-r}^{i+r} \theta_{j}}+\bar{\text{cov}\left[ \left( v_{j}^{b} \right)_{k},\left( v_{j'}^{b} \right)_{k} \right]} (S7)$$

where

$$\bar{\left( \sigma_{i}^{\text{bio}} \right)_{k}^{2}}=\frac{1}{\sum_{j=i-r}^{i+r} \theta_{j}}\sum_{j=i-r}^{i+r} \theta_{j}\left( \sigma_{j}^{\text{bio}} \right)_{k}^{2} , (S8)$$

gives the average variance over biological replicates under experimental condition $k$ of the expression levels for the genes inside the window centered at $i$^th^ position of the ordered list. The second term in Eq.(S7) contains the average over the covariance for the biological variation of all pairs of genes inside the window, that is,

$$\bar{\text{cov}\left[ \left( v_{j}^{b} \right)_{k},\left( v_{j^{'}}^{b} \right)_{k} \right]} = \left( S9 \right)$$

$$\frac{2}{\left( \sum_{j=i-r}^{i+r} \theta_{j} \right)^{2}}\sum_{j=i-r}^{i+r-1} \sum_{j^{'}=j+1}^{i+r} \theta_{j}\theta_{j^{'}}\left[ \left\langle\left( v_{j}^{b} \right)_{k} \left( v_{j^{'}}^{b} \right)_{k} \right\rangle-\left\langle\left( v_{j}^{b} \right)_{k} \right\rangle\left\langle\left( v_{j^{'}}^{b} \right)_{k} \right\rangle\right] .$$

The biological meaning of this term may be clarified from the consideration of two limit possibilities. First, assume that these covariances are zero, implying that biological samples may have the expression of the genes inside the window varying independently of one another. In this case

$$\left[ \left\langle\left( V_{i}^{b} \right)_{k}^{2} \right\rangle-\left\langle\left( V_{i}^{b} \right)_{k} \right\rangle^{2} \right]= \frac{\bar{\left( \sigma_{i}^{\text{bio}} \right)_{k}^{2}}}{\sum_{j=i-r}^{i+r} \theta_{j}} , (S10)$$

representing a strong decrease in the biological variance, as compared to the case when $r=0$. However, the other limit case happens when the biological variations are the same for all genes inside the window, that is, $\left( v_{j}^{b} \right)_{k}= \left( v_{j'}^{b} \right)_{k}$ for all pairs $(j,j^{'})$. In this case,

$$\left[ \left\langle\left( V_{i}^{b} \right)_{k}^{2} \right\rangle-\left\langle\left( V_{i}^{b} \right)_{k} \right\rangle^{2} \right]= \bar{\left( \sigma_{i}^{\text{bio}} \right)_{k}^{2}} . (S11)$$

This last situation may happen when all genes in the window are regulated by the same transcription factor. The stochastic contribution for transcriptogram variance in Eq.(S6) is always

$$\left[ \left\langle\left( R_{i}^{b,a} \right)_{k}^{2} \right\rangle-\left\langle\left( R_{i}^{b,a} \right)_{k} \right\rangle^{2} \right]= \frac{\bar{\left( \sigma_{i}^{\text{tech}} \right)_{k}^{2}}}{\sum_{j=i-r}^{i+r} \theta_{j}} , (S12)$$

since the stochastic noise is assumed uncorrelated. Eq.(S6) may then be rewritten as

$$\left\langle\left( T_{i}^{b,a} \right)_{k}^{2} \right\rangle-\left\langle\left( T_{i}^{b,a} \right)_{k} \right\rangle^{2}= \frac{\bar{\left( \sigma_{i}^{\text{total}} \right)_{k}^{2}}}{\sum_{j=i-r}^{i+r} \theta_{j}}+\bar{\text{cov}\left[ \left( v_{j}^{b} \right)_{k},\left( v_{j'}^{b} \right)_{k} \right]} . (S13)$$

The variances given in Eqs. (S5) and (S12), are relevant in the significance assessment of the difference in expression of samples under different experimental conditions, since these procedures usually involve the comparison of the difference in average expression of two conditions with the sum of the standard deviations of each conditions. For expression values, a pertinent quantity is the ratio $\left( \epsilon_{i} \right)_{1,2}$, given as

$$\left( \epsilon_{i} \right)_{1,2}=\frac{\left\langle\left( t_{i}^{b,a} \right)_{2}-\left( t_{i}^{b,a} \right)_{1} \right\rangle\sqrt{n_{a}n_{b}}}{\left( \sigma_{i}^{\text{total}} \right)_{1}+\left( \sigma_{i}^{\text{total}} \right)_{2}} , (S14)$$

that measures the difference between the average over replicates of transcriptions of conditions 1 and conditions 2 in unities given by the sum of two standard deviations of the mean. (Here we assume both conditions to have the same number of replicates, for simplicity.) The same quantity, for the transcriptogram is

$$\left( Z_{i} \right)_{1,2}=\frac{\left\langle\left( T_{i}^{b,a} \right)_{2}-\left( T_{i}^{b,a} \right)_{1} \right\rangle\sqrt{n_{a}n_{b}}}{\sqrt{\frac{\bar{\left( \sigma_{i}^{\mathrm{total}} \right)_{2}^{2}}}{\sum_{j=i-r}^{i+r} \theta_{j}}+ \bar{\text{cov}\left[ \left( v_{j}^{b} \right)_{2},\left( v_{j^{'}}^{b} \right)_{2} \right]}}+ \sqrt{\frac{\bar{\left( \sigma_{i}^{\mathrm{total}} \right)_{1}^{2}}}{\sum_{j=i-r}^{i+r} \theta_{j}}+ \bar{\text{cov}\left[ \left( v_{j}^{b} \right)_{1},\left( v_{j^{'}}^{b} \right)_{1} \right]}}} ,$$

$$(S15)$$

meaning that, provided $\left\langle\left( T_{i}^{b,a} \right)_{2}-\left( T_{i}^{b,a} \right)_{1} \right\rangle$ decreases less that the denominator, transcriptograms may enhance the sensitivity of microarray measures. As already mentioned above, this may happen when the biological variance is not correlated over the window, that is, when

$$\bar{\text{cov}\left[ \left( v_{j}^{b} \right)_{k},\left( v_{j'}^{b} \right)_{k} \right]}\ll\frac{\bar{\left( \sigma_{i}^{\mathrm{total}} \right)_{k}^{2}}}{\sum_{j=i-r}^{i+r} \theta_{j}}, (S16)$$

for both conditions $k$, which yields

$$\left( Z_{i} \right)_{1,2}=\frac{\left\langle\left( T_{i}^{b,a} \right)_{2}-\left( T_{i}^{b,a} \right)_{1} \right\rangle\sqrt{n_{a}n_{b}}}{\left( \sigma_{i}^{\text{total}} \right)_{1}+\left( \sigma_{i}^{\text{total}} \right)_{2}}\sqrt{\sum_{j=i-r}^{i+r} \theta_{j}} . (S17)$$

Comparing Eqs. (S14) and (S17), for the transcriptogram procedure to enhance sensitivity, $\left\langle\left( T_{i}^{b,a} \right)_{2}-\left( T_{i}^{b,a} \right)_{1} \right\rangle$ should decrease slower than $\left( \sum_{j=i-r}^{i+r} \theta_{j} \right)^{-\frac{1}{2}}$, that is, slower than $\left( 2r+1 \right)^{-\frac{1}{2}}$, for windows where all genes are represented in the microarray. This implies that the different metabolic states representing each condition impose correlated modifications on the expression on the genes located inside the ordered list window, what should be expected when the window is enriched with genes of biological functions that are differently affected in both conditions.

All results depend on the chosen ordering for the gene lists as well as on the size of the window.


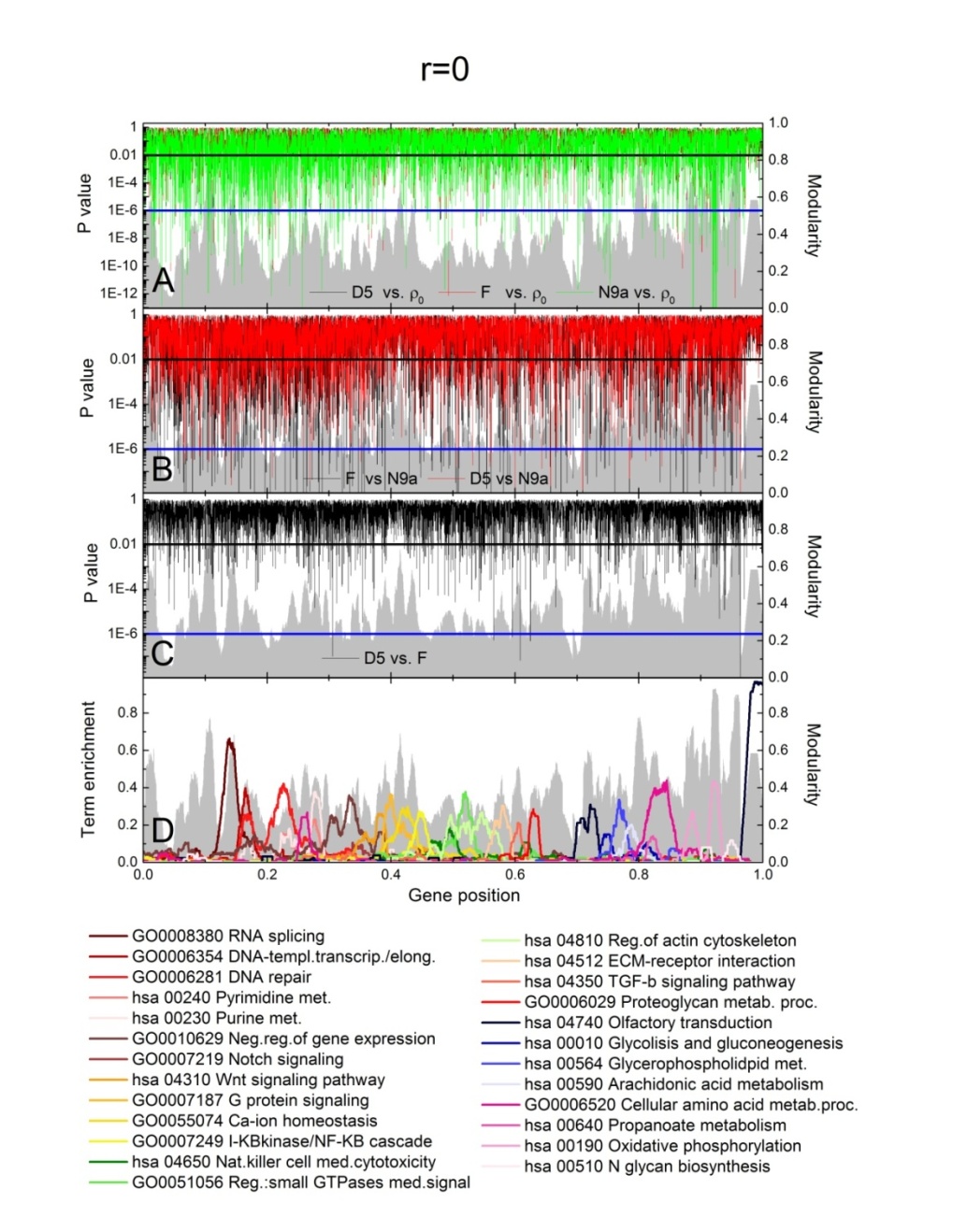


Figure S 1. *P*-values of transcriptogram means for all gene positions in $\boldsymbol{\alpha=1}$ ordering and $\boldsymbol{r=0}$ for A) F, D5, and N9a cybrids compared to $\boldsymbol{\rho}_{\boldsymbol{0}}$ cell line; B) F and D5 cybrids compared to N9a cybrid; C) D5 cybrid compared to F cybrid. D) Selected gene ontology terms and KEGG pathways profiles for $\boldsymbol{r=80}$ . Gray background is the window modularity profile for $\boldsymbol{r=80}$ , to guide the eye. The horizontal, black line in panels A-C represents *P*=0.01, while the blue line at *P*=0.01/9684 corresponds to the Bonferroni correction for multiple hypotheses testing.

|  | | FDR Radius = 0 | | | | | | |
| --- | --- | --- | --- | --- | --- | --- | --- | --- |
| P value | N9a vs ρ_0_ | | F vs ρ_0_ | D5 vs ρ_0_ | F vs N9a | D5 vs N9a | D5 vs F |  |
| 0.1 | 0.145389 | | 0.171001 | 0.139366 | 0.157776 | 0.295737 | 0.548948 |  |
| 0.05 | 0.091211 | | 0.110416 | 0.080086 | 0.098791 | 0.269759 | 0.464294 |  |
| 0.01 | 0.038148 | | 0.056859 | 0.025014 | 0.038567 | 0.278242 | 0.326013 |  |
| 0.005 | 0.025381 | | 0.045119 | 0.016431 | 0.025939 | 0.27055 | 0.617382 |  |
| 0.001 | 0.01015 | | 0.024616 | 0.005609 | 0.009346 | 0.161983 | ---- |  |

Table S 1 False discovery rate (FDR) calculated at different $\boldsymbol{P}$values for $\boldsymbol{\alpha}\boldsymbol{=1}$ ordering and $\boldsymbol{r}\boldsymbol{=0}$ transcriptograms for different pairs of conditions.


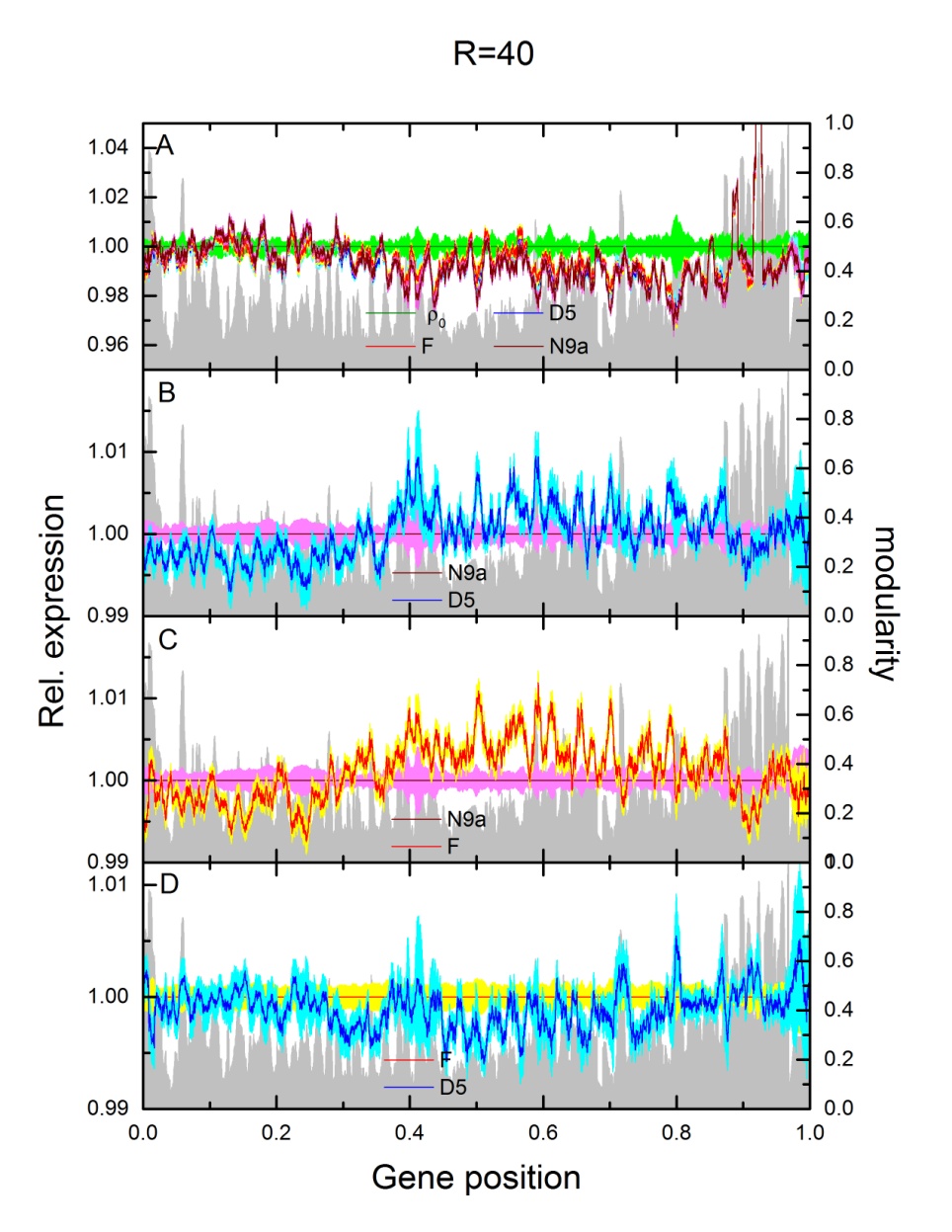


Figure S 2 Relative average transcriptograms ($\boldsymbol{\alpha}\boldsymbol{=1,}\boldsymbol{r}\boldsymbol{=40)}$ with colored regions standing for the respective standard errors for A) N9a, F and D5 cybrids relative to the $\boldsymbol{\rho}_{\boldsymbol{0}}$ cell line; B) D5 cybrid relative to N9a cybrid transcriptograms; C) F cybrid relative to N9a cybrid; D) D5 cybrid relative to F cybrid.


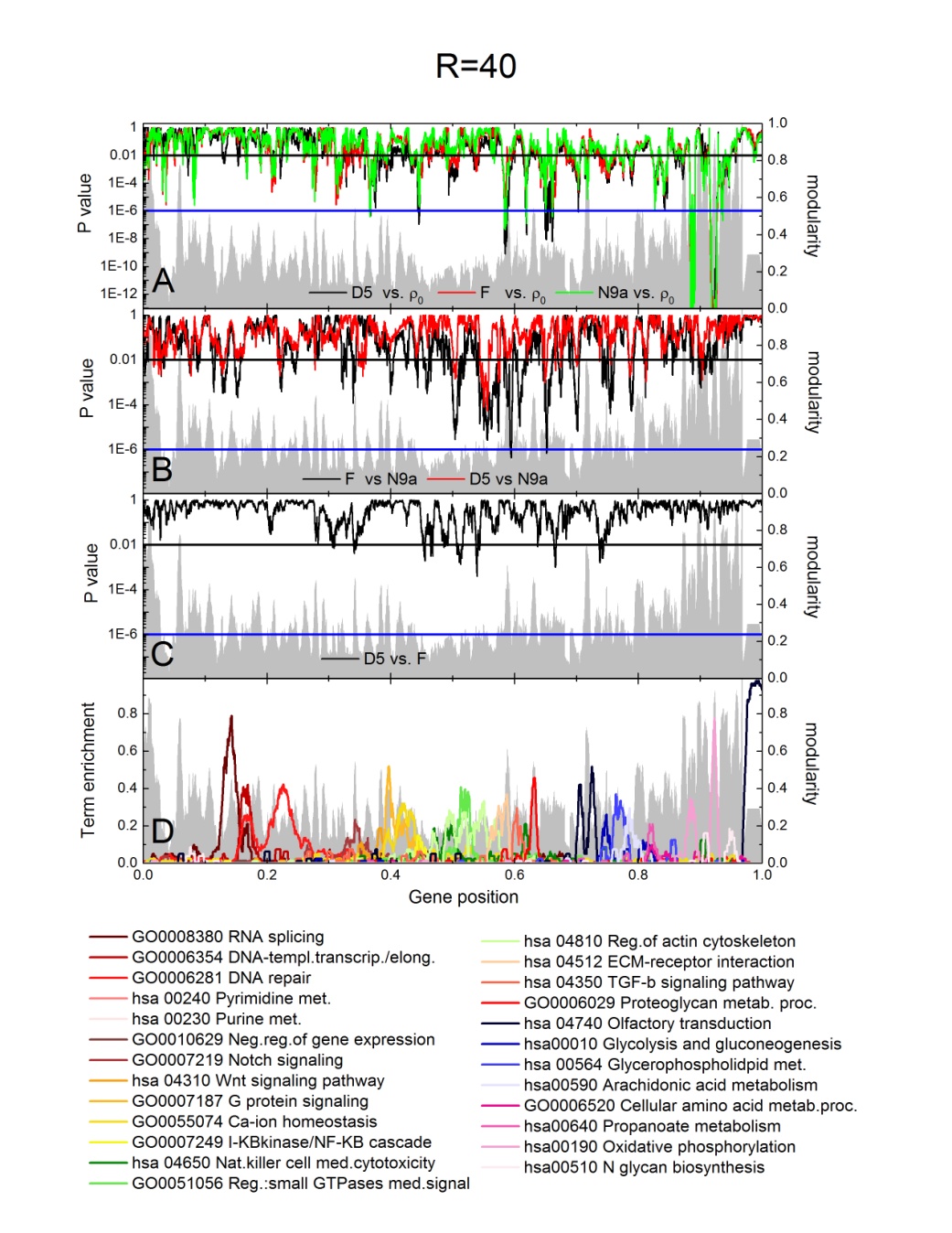


Figure S 3 $\boldsymbol{P}$-values of transcriptogram means for all gene positions in $\boldsymbol{\alpha}\boldsymbol{=}\boldsymbol{1}$ ordering and $\boldsymbol{r}\boldsymbol{=4}\boldsymbol{0}$ for A) F, D5 and N9a cybrids compared to $\boldsymbol{\rho}_{\boldsymbol{0}}$ cell line; B) F and D5 cybrids compared to N9a cybrid; C) and D5 cybrid compared to F cybrid. D) Selected gene ontology terms and KEGG pathways profiles for $\boldsymbol{r}\boldsymbol{=4}\boldsymbol{0}$. Gray background is the window modularity profiles for $\boldsymbol{r}\boldsymbol{=4}\boldsymbol{0}$, to guide the eye. The horizontal, black line in panels A-C represents $\boldsymbol{P}\boldsymbol{=}\boldsymbol{0}\boldsymbol{.}\boldsymbol{01}$, while the horizontal blue line at P=0.01/9684 corresponds to the Bonferroni correction for multiple hypotheses testing.

| FDR R=40 | | | | | | | |
| --- | --- | --- | --- | --- | --- | --- | --- |
| P value | N9a vs ρ_0_ | F vs ρ_0_ | D5 vs ρ_0_ | F vs N9a | D5 vs N9a | D5 vs F |  |
| 0.1 | 0.154355 | 0.154739 | 0.186043 | 0.297024 | 0.175157 | 0.564702 |  |
| 0.05 | 0.096303 | 0.091315 | 0.127348 | 0.223973 | 0.106359 | 0.417623 |  |
| 0.01 | 0.039759 | 0.029702 | 0.064727 | 0.134764 | 0.036195 | 0.286187 |  |
| 0.005 | 0.025785 | 0.018259 | 0.049799 | 0.12261 | 0.0216 | 0.260662 |  |
| 0.001 | 0.010344 | 0.006258 | 0.026114 | 0.096852 | 0.006697 | 1.3366 |  |
|  |  |  |  |  |  |  |  |
|  |  |  |  |  |  |  |  |

Table S 2 - False discovery rate (FDR) calculated at different $\boldsymbol{P}$values for $\boldsymbol{\alpha}\boldsymbol{=1}$ ordering and $\boldsymbol{r}\boldsymbol{=40}$ transcriptograms for different pairs of conditions.

Figure S 4. Expression levels for 6 technical replicates for $\boldsymbol{\rho}_{\boldsymbol{0}}$ samples. The variation for the expression between replicates is lesser than the variation between different genes.

Figure S 5 Contrast-to-noise (upper panel) and signal-to-noise (lower panel) calculated for calculated for the technical replicates of ρ_0_ cell line. Observe that while contras-to-noise ratio goes decreases as r increases, but signal-to-noise ratio is a monotonically increasing function of r.


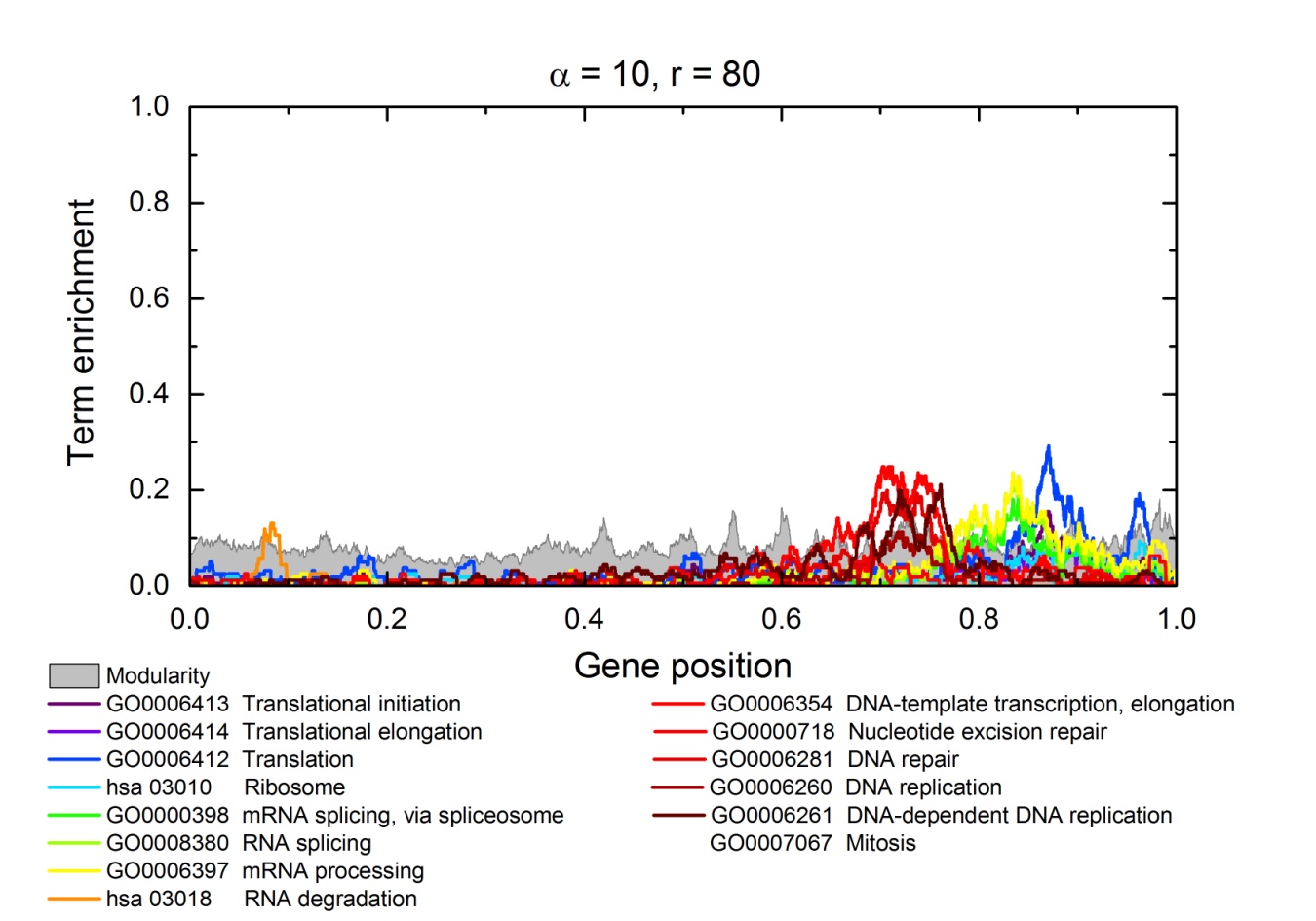


Figure S 6. The same panel as Figure 2 A in the main text, but here for an ordering with $\boldsymbol{\alpha=10.}$ Window modularity for$\boldsymbol{r=80}$, shown as a gray solid profile, is greatly reduced. The same GO terms as in Figure 2 A appear as lower, more spread peaks, now concentrated at the right end of the figure.

|  |  |  |  |  |  |  |
| --- | --- | --- | --- | --- | --- | --- |


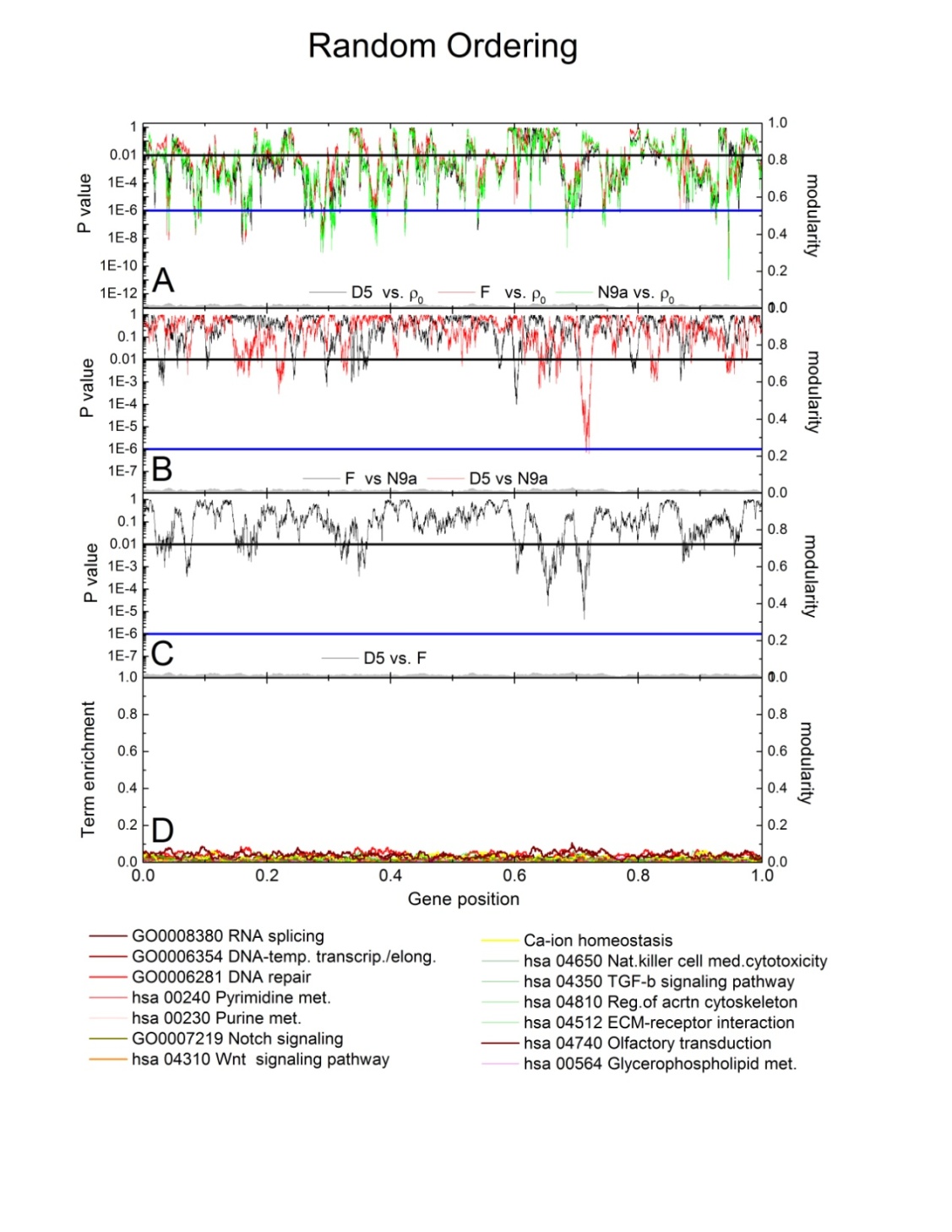


Figure S 7 $\boldsymbol{P}$-values of transcriptogram means for all gene positions in a random ordering and $\boldsymbol{r}\boldsymbol{=80}$for A) F, D5 and N9a cybrids compared to $\boldsymbol{\rho}_{\boldsymbol{0}}$ cell line; B) F and D5 cybrids compared to N9a cybrid; C) and D5 cybrid compared to F cybrid. D) Selected gene ontology terms and KEGG pathways profiles. Gray background is the window modularity profiles, as in Fig. 7 of main text, but here the values are very low. The horizontal, black line in panels A-C represents $\boldsymbol{P}\boldsymbol{=0.01}$, while the horizontal blue line at P=0.01/9684 corresponds to the Bonferroni correction for multiple hypotheses testing.

| FDR Random ordering | | | | | | | |
| --- | --- | --- | --- | --- | --- | --- | --- |
| P value | N9a vs ρ_0_ | F vs ρ_0_ | D5 vs ρ_0_ | F vs N9a | D5 vs N9a | D5 vs F |  |
| 0.1 | 0.138121 | 0.141773 | 0.117864 | 0.489981 | 0.328207 | 0.228864 |  |
| 0.05 | 0.085209 | 0.08935 | 0.060636 | 0.389165 | 0.222059 | 0.157523 |  |
| 0.01 | 0.031283 | 0.036053 | 0.017806 | 0.174407 | 0.089972 | 0.054758 |  |
| 0.005 | 0.020932 | 0.024829 | 0.011223 | 0.15447 | 0.066378 | 0.034355 |  |
| 0.001 | 0.007823 | 0.009882 | 0.003201 | 0.199432 | 0.030125 | 0.011809 |  |
|  |  |  |  |  |  |  |  |

Table S 3 False discovery rate (FDR) calculated at different $\boldsymbol{P}$values for a random ordering and $\boldsymbol{r}\boldsymbol{=}$80 transcriptograms for different pairs of conditions.

**References**

|  |  |  |  |  |  |  |
| --- | --- | --- | --- | --- | --- | --- |

Irizarry RA, Hobbs B, Collin F, Beazer-Barclay YD, Antonellis KJ, et al. (2003) Exploration, normalization, and summaries of high density oligonucleotide array probe level data. *Biostatistics* 4: 249–264.
